# Biomechanical stimulation promotes blood vessel growth despite VEGFR-2 inhibition

**DOI:** 10.1186/s12915-023-01792-y

**Published:** 2023-12-10

**Authors:** Bronte Miller Johnson, Allison McKenzie Johnson, Michael Heim, Molly Buckley, Bryan Mortimer, Joel L. Berry, Mary Kathryn Sewell-Loftin

**Affiliations:** 1 Department of Biomedical Engineering, University of Alabama at Birmingham, 1824 6th Avenue South, Wallace Tumor Institute, Room 630A, Birmingham, AL 35294 USA; 2 Heersink School of Medicine, University of Alabama at Birmingham, Birmingham, AL 35233 USA; 3 O’Neal Comprehensive Cancer Center, University of Alabama at Birmingham, Birmingham, AL 35233 USA

**Keywords:** VEGFR-2, VEGF, Angiogenesis, Mechanobiology, Strain, Tumor microenvironment, Endothelial cells

## Abstract

**Background:**

Angiogenesis, or the growth of new vasculature from existing blood vessels, is widely considered a primary hallmark of cancer progression. When a tumor is small, diffusion is sufficient to receive essential nutrients; however, as the tumor grows, a vascular supply is needed to deliver oxygen and nutrients into the increasing mass. Several anti-angiogenic cancer therapies target VEGF and the receptor VEGFR-2, which are major promoters of blood vessel development. Unfortunately, many of these cancer treatments fail to completely stop angiogenesis in the tumor microenvironment (TME). Since these therapies focus on the biochemical activation of VEGFR-2 via VEGF ligand binding, we propose that mechanical cues, particularly those found in the TME, may be a source of VEGFR-2 activation that promotes growth of blood vessel networks even in the presence of VEGF and VEGFR-2 inhibitors.

**Results:**

In this paper, we analyzed phosphorylation patterns of VEGFR-2, particularly at Y1054/Y1059 and Y1214, stimulated via either VEGF or biomechanical stimulation in the form of tensile strains. Our results show prolonged and enhanced activation at both Y1054/Y1059 and Y1214 residues when endothelial cells were stimulated with strain, VEGF, or a combination of both. We also analyzed Src expression, which is downstream of VEGFR-2 and can be activated through strain or the presence of VEGF. Finally, we used fibrin gels and microfluidic devices as 3D microtissue models to simulate the TME. We determined that regions of mechanical strain promoted increased vessel growth, even with VEGFR-2 inhibition through SU5416.

**Conclusions:**

Overall, understanding both the effects that biomechanical and biochemical stimuli have on VEGFR-2 activation and angiogenesis is an important factor in developing effective anti-angiogenic therapies. This paper shows that VEGFR-2 can be mechanically activated through strain, which likely contributes to increased angiogenesis in the TME. These proof-of-concept studies show that small molecular inhibitors of VEGFR-2 do not fully prevent angiogenesis in 3D TME models when mechanical strains are introduced.

**Supplementary Information:**

The online version contains supplementary material available at 10.1186/s12915-023-01792-y.

## Background

Improving our understanding of the mechanobiology of the tumor microenvironment (TME), including how biomechanical forces drive tumor progression and blood vessel growth, will enable the development of novel anti-cancer therapeutic strategies. When a tumor is small, oxygen and other nutrients diffuse throughout the growing mass; however, once the tumor reaches a size larger than about 100–200 µm in radius, a decrease in oxygen creates a hypoxic environment which promotes angiogenesis [1, 2]. Angiogenesis is defined as the growth of new blood vessels from an existing vascular network and is considered a hallmark of tumor progression [3]. The pro-angiogenic pathways involved in tumor progression have long been targeted as a method of treating cancer, with the thought that limiting blood vessel growth would deprive the tumor of sufficient oxygen or nutrients to continue growing [4]. Vasculogenesis, or the assembly of blood vessels de novo, is critical for development, and angiogenesis is involved in other processes such as wound healing [5, 6]. In response to a hypoxic stimulus, cancer cells begin secreting vascular endothelial growth factor (VEGF), which then binds to VEGF receptor-2 (VEGFR-2) on nearby vascular endothelial cells (ECs) [7, 8]. The binding of VEGF to the receptor causes a breakdown of the vessel basement membrane through internalization of VE-cadherin, which allows certain ECs to sprout off of the pre-existing vasculature and migrate towards the VEGF gradient [7, 9, 10]. The migratory ECs leading the growing vessel are referred to as tip cells and express high levels of VEGFR-2, which guides the proliferating stalk cells towards the tumor [11]. The precise manner in which some cells become tip cells, while other ECs remain as stalk cells, is unclear, although Notch1 signaling is implicated [12]. More recently, metabolic regulation has been implicated in defining tip and stalk cell behaviors [13]. Furthermore, the phenotypic shift that occurs during the formation of a tip cell is reminiscent of epithelial-to-mesenchymal transformation, a process which can be partially stimulated through tensile strains [14, 15].

While there are many members in the VEGF and VEGFR family, the primary promotors of angiogenesis are VEGF and VEGFR-2 [16]. While VEGFR-1 and VEGFR-3 are closely related to VEGFR-2, these receptors are involved in anti-angiogenic regulation and lymphangiogenesis, respectively [17]. VEGFR-2 remains the primary pro-angiogenic promotor, and it is made up of an extracellular ligand-binding domain and an intracellular tyrosine kinase domain [16, 18]. When the ligand VEGF binds to VEGFR-2, the receptor dimerizes and transphosphorylates tyrosine residues on the intracellular domain [19]. Some prominent residues involved in VEGFR-2 signaling are Y1054/Y1059, which are necessary for complete kinase activity, and Y1175 which is associated with ERK1/2 phosphorylation [20, 21]. Finally, the Y1214 residue also regulates ERK1/2 and the Akt pathway [22, 23]. Notably, this Y1214 residue was shown to express prolonged phosphorylation when VEGFR-2 is presented with matrix-bound VEGF compared to soluble VEGF [24]. The anchorage of the ligand may alter the mechanical force that VEGFR-2 is exposed to, resulting in this change in phosphorylation patterns. We hypothesize that ECs exposed to mechanical strain may exhibit increased or prolonged VEGFR-2 phosphorylation and that mechanical strains promote angiogenesis even in the presence of VEGFR-2 inhibitors.

Previous investigations of mechanical regulation of the VEGFR-2 pathway have been limited due to model constraints. For mouse studies, knockout of the *vegfr-2* gene is embryonically lethal, leading to a rise in conditional knock-outs and transgenic models to target this receptor and for the development of anti-angiogenic treatment strategies [25]. While these approaches have been useful in developing clinical therapies that target VEGF/VEGFR-2, the biomechanical microenvironment of mice is dramatically different from humans. The discrepancy in microenvironments and mechanical forces may be partially responsible for the limited efficacy of anti-VEGFR-2-based therapies in several types of cancer, including breast cancer [25, 26]. Many of these therapies utilize small molecules or monoclonal antibodies and focus on preventing VEGF from binding to VEGFR-2 or by binding to VEGFR-2 to prevent activation which then inhibits subsequent downstream signaling. In patients, anti-VEGF/VEGFR-2 therapies lead to modest changes in disease progression with minimal increases in overall survival [26–28]. In fact, bevacizumab, a recombinant monoclonal antibody against VEGF, was pulled from recommendation by the FDA for breast cancer patients due to its limited efficacy [29]. Furthermore, the limitations of anti-VEGF strategies may also be due in part to our limited understanding of mechanical regulation of VEGFR-2 activation and downstream signaling. Within tissues, ECs can experience a combination of compressive forces, tensile strains, and/or shear stresses generated by flow either inside a vessel or from interstitial flow [30]. These forces can alter not only VEGFR-2 signaling but other key signaling pathways related to cytoskeletal organization, migration, and proliferation [31–33]. Previous research has demonstrated that a variety of physical forces such as matrix stiffness and shear stress can increase VEGFR-2 and subsequent downstream activity, but few studies have focused on the effects that mechanical strain has on VEGFR-2 phosphorylation and angiogenesis [34–36].

The tumor microenvironment (TME) is mechanically dysregulated compared to normal tissue with respect to numerous components including matrix properties, interstitial fluid flow, and both compression and tensile forces. First, the extracellular matrix (ECM) is stiffer due to an increase in collagen produced by stromal cells such as cancer-associated fibroblasts (CAFs) [37–39]. Furthermore, the interstitial fluid pressure is higher in the TME due to leaky vasculature caused by degradation of the blood vessel basement membrane [40]. Fluid flow within the tumor blood vessels is often disturbed because the vasculature is tortuous with sections of blood stasis or high shear stress [41, 42]. In the TME, CAFs are also known to be mechanically active, with higher contractility that generates larger matrix deformations or strains compared to normal fibroblasts [43, 44]. These CAFs thus induce strain on the matrix surrounding ECs, causing these ECs to experience increased strain compared to ECs surrounded by normal fibroblasts. Moreover, in the TME since fibroblasts often adhere to vasculature to behave as pericyte-like cells that support actively growing blood vessels, the high contractility of CAFs would induce pulling or stretching of blood vessels, resulting in strain on ECs. Understanding how mechanical forces, particularly strain, in the TME impact VEGFR-2 activation is important because they may be causing VEGF-independent phosphorylation of this receptor, resulting in increased angiogenesis even in the presence of anti-angiogenic therapies. Recent work has focused on developing 3D ex vivo models of the human TME by using microfluidic devices to generate microtissues with multiple cell types and functional vascular networks [45–50]. These microphysiological systems leverage control over biophysical cues, including matrix composition or interstitial flow patterns to investigate cellular behaviors in co-culture models that can include tumor cells, ECs, and other stromal components. The use of such TME models permits advanced investigations of mechanobiological signaling and processes such as angiogenesis.

## Results

### VEGFR-2 inhibition alters VEGFR-2 expression and phosphorylation

**Fig. 1:**
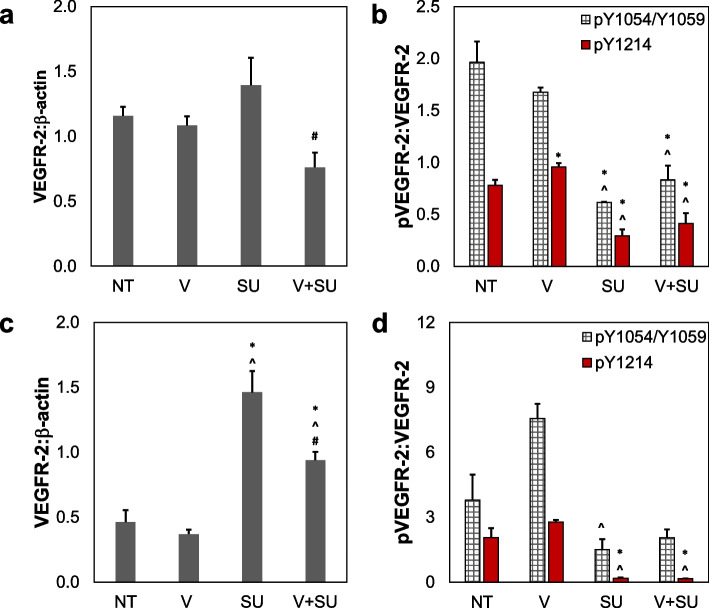
VEGFR-2 expression and phosphorylation patterns in HMECs and HUVECs with VEGF or SU5416. **a** Quantification for protein levels of VEGFR-2 in HMECs treated with either no treatment (NT) control media, 25 ng/mL VEGF (V), 3 µM SU5416 (SU), or both VEGF and SU5416 (V + SU) for 72 h. **b** Quantified Western blot analyses for phosphorylated VEGFR-2 at Y1054/Y1059 and Y1214 normalized to total VEGFR-2 values for each HMEC sample. **c** Quantification for protein levels of VEGFR-2 in HUVECs treated with either no treatment (NT) control media, 25 ng/mL VEGF (V), 3 µM SU5416 (SU), or both VEGF and SU5416 (V + SU) for 72 h. **d** Quantified Western blot analyses for phosphorylated VEGFR-2 at Y1054/Y1059 and Y1214 normalized to total VEGFR-2 values for each HUVEC sample. For all images, Western blot quantifications were normalized to β-actin, while phosphorylation quantifications being double-normalized to total VEGFR-2. *p < 0.05 compared to NT, ^*p* < 0.05 compared to V, #*p* < 0.05 compared to SU. Data shown as average + SEM, *n* = 6 for a and c, *n* = 3 for **b** and **d**. Group **d** was compared with Kruskal–Wallis test, followed by post hoc Dunn’s tests. Groups **a–c** were compared with ANOVA, followed by post hoc Tukey HSD tests

In initial studies, both HMECs (human microvascular endothelial cells) and HUVECs (human umbilical vein endothelial cells) were treated with combinations of VEGF and the VEGFR-2 inhibitor SU5416 to analyze how these treatments affect both VEGFR-2 expression and phosphorylation. In HMECs, treatment with both VEGF and SU5416 demonstrates a significant decrease in receptor expression compared to the strain treatment group (Fig. 1a, Additional File 1: Fig. S1). Western blot results for both pY1054/Y1059 and pY1214 of VEGFR-2 in HMECs show a significant decrease in phosphorylation of both residues when SU5416 was added compared to NT or VEGF only, while the combination of VEGF and SU5416 treatment partially negates this impact (Fig. 1b). In contrast, VEGFR-2 protein levels in HUVECs are increased significantly in samples that received SU5416 treatment compared to all other treatments and slightly higher for the combination of VEGF and SU5416 versus NT and VEGF only groups (Fig. 1c). Similarly, phosphorylation of Y1054/1059 and Y1214 tyrosine residues show a significant decrease with the addition of SU5416 (Fig. 1d). Overall, HMECs and HUVECs demonstrate similar trends of decreased phosphorylation of VEGFR-2 when treated with the inhibitor. The addition of VEGF may partially rescue this reduction in phosphorylation. These studies were completed without strain stimulation to generate a baseline understanding of phosphorylation patterns in VEGFR-2 with respect to SU5416 treatment.

### Uniaxial strain with VEGF causes increased VEGFR-2 phosphorylation in ECs

**Fig. 2:**
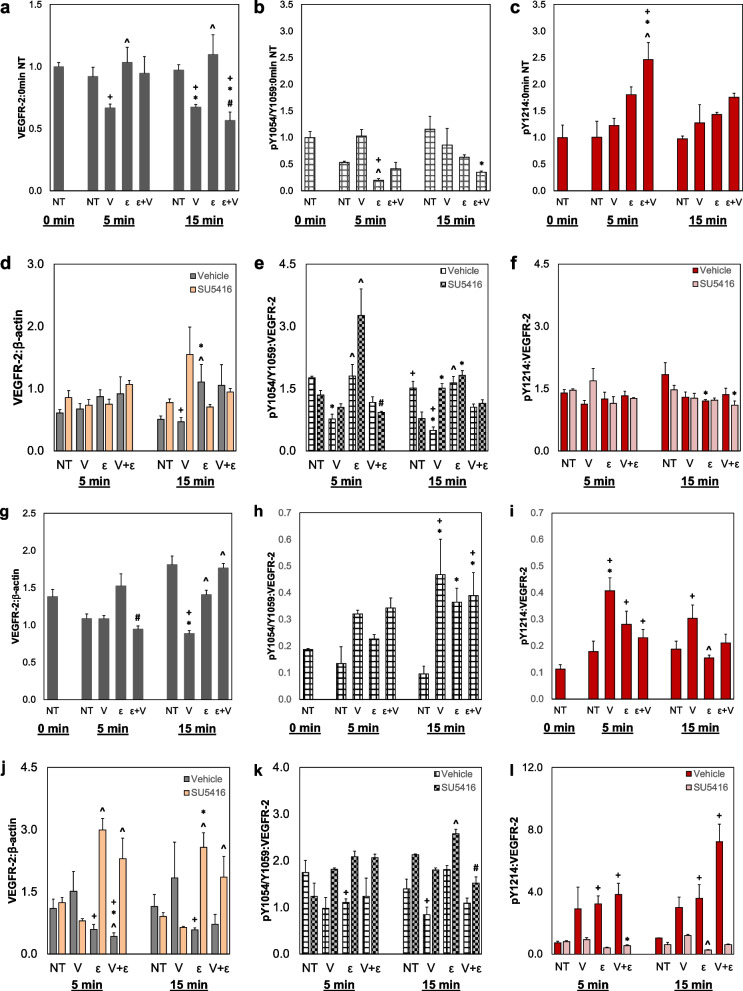
Strain treatment alters VEGFR-2 expression and phosphorylation patterns in HMECs and HUVECs. **a** HMECs were grown on Flexcell plates and subjected to control (NT) conditions without strain and VEGF, VEGF only (25 ng/mL, V), strain only (ε), or strain plus VEGF (V + ε) for 5 or 15 min. Western blots were performed to analyze total VEGFR-2, **b** pY1054/Y1059, or **c** pY1214 levels relative to the 0 min, NT control groups. **d** HMECs were treated with combinations of strain, VEGF (25 ng/mL), SU5416 (3 µM), and a vehicle control then stained for total VEGFR-2, **e** pY1054/Y1059, and **f** pY1214. **g**–**l** Quantification of Western blots for total or phosphorylated VEGFR-2 in HUVECs with same treatment groups outlined above; however, pY1054/Y1059 was quantified for the 160 kDa cleavage product due to partial absence of the mature product in the blot for part **k**. Western blot quantifications were normalized to β-actin, with phosphorylation quantifications being double-normalized to total VEGFR-2. For **a**–**c** and **g**–**i**, + *p* < 0.05 versus 0 min NT, * *p* < 0.05 compared to NT at same time point, ^ *p* < 0.05 versus V at same time point, # *p* < 0.05 compared to **e** at same time point. For **d**–**f** and **j**–**l**, + *p* < 0.05 versus SU5416 with same treatment, * *p* < 0.05 compared to NT at same time point, ^ *p* < 0.05 versus V at same time point, # *p* < 0.05 compared to ε at same time point. Data shown as average + SEM, *n* = 6 for **a**, **d**, **g**, and **j**, *n* = 3 for all other samples. Groups **a**, **d**–**f**, **h**–**j**, and **l** were compared with Kruskal–Wallis test, followed by post hoc Dunn’s tests. Groups **b**, **c**, **g**, and **k** were compared with ANOVA, followed by post hoc Tukey HSD tests

Phosphorylation and total VEGFR-2 levels were measured for HMECs and HUVECs stimulated with VEGF and/or strain using the Flexcell system. In HMECs, results show that VEGF (V) treatments at both 5 and 15 min significantly decrease total VEGFR-2 by ~ 25% compared to no treatment (NT) groups, while strain (ε) does not appear to have a significant effect (Fig. 2a, Additional File 1: Fig. S2). For HMECs, strain at 5 min decreases pY1054/Y1059 levels compared to NT and VEGF only; however, strain plus VEGF shows a significant increase in pY1214 at 5 min compared to NT and VEGF only (Fig. 2b, c). The increases in pY1214 levels in HMECs due to strain stimulation are on the order of 1.5–2.5 × compared to NT samples at the same time points. Additionally, both ECs were treated with 5 or 15 min strain, VEGF, and the inhibitor SU5416 or a vehicle control. HMECs with both VEGF and SU5416 demonstrate a significant increase in total VEGFR-2 compared to only VEGF-treated samples, which is not seen when strain was added (Fig. 2d). Regarding phosphorylation, strain with SU5416 treatment shows a significant increase in pY1054/Y1059 compared to groups with VEGF and SU5416; however, pY1214 varies very little between treatment groups (Fig. 2e, f).

On the other hand, HUVEC samples show a significant increase in total VEGFR-2 for strain and strain plus VEGF groups at 15 min compared to only VEGF at 15 min (Fig. 2g). Furthermore, 15 min of strain treatment shows a significant nearly fourfold increase of in pY1054/Y1059 levels compared to NT controls, but no similar increase is observed in pY1214 levels (Fig. 2h). HUVEC data also indicate a 2 × increase in pY1214 at 5 min with strain, which decreases over time by 15 min (Fig. 2i). For the groups with the inhibitor, HUVECs receiving SU5416 displays significantly increased in total VEGFR-2 at both 5 and 15 min (Fig. 2j). Due to this change in total VEGFR-2, we also analyzed phosphorylated VEGFR-2 normalized to only β-actin rather than double normalizing to total VEGFR-2 (Additional File 1: Fig. S3). Phosphorylation at Y1054/Y1059 in HUVECs shows a significant increase when treated with strain and SU5416 compared to VEGF and strain when normalized to total VEGFR-2; however, pY1214 is diminished with the addition of SU5416 regardless of VEGF or strain treatment. Still, in the vehicle groups, cells treated with strain display increased phosphorylation compared to NT groups (Fig. 2k, l).These studies indicate that strain alone can affect the expression and phosphorylation at different tyrosine residues of different EC types, that these patterns may be partially conserved between cell types, and that SU5416 inhibition of VEGFR-2 does not fully block phosphorylation events.

### Mechanical strain alters Src expression

**Fig. 3:**
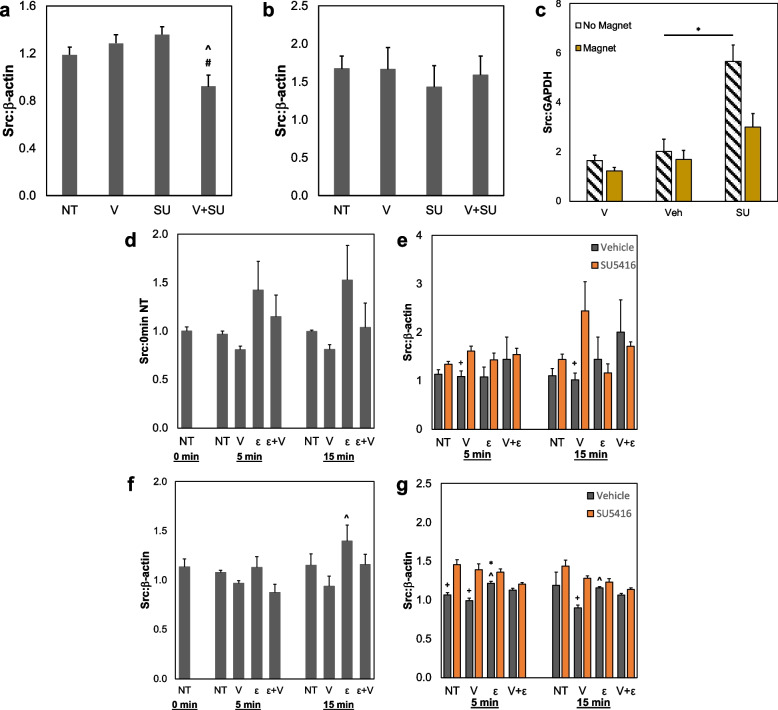
Effects of strain and VEGFR-2 activation or inhibition on Src activity. **a** HMECs and **b** HUVECs were treated with control, no treatment (NT) media, 25 ng/mL VEGF (V), 3 µM SU5416 (SU), or both VEGF and SU5416 (V + SU) for 72 h. Western blot analysis for total levels of Src was performed and quantified. *p < 0.05 versus NT, ^ *p* < 0.05 versus V, # *p* < 0.05 versus SU. **c** HUVECs in 3D fibrin gels with magnetic beads were treated with 25 ng/mL VEGF (V), DMSO as a vehicle control (Veh), or 3 µM SU5416 (SU) for 72 h. Some samples received no external magnetic stimulation (No Magnet) while other samples were cultured above a rotating magnetic field (Magnet) to generate matrix distortions around the cells. Src was analyzed via Western blot and normalized to GAPDH as a loading control. * *p* < 0.05. **d** HMECs were subjected to control (NT) conditions without strain and VEGF, VEGF only (25 ng/mL, V), strain only (ε), or strain plus VEGF (V + ε) for 5 or 15 min before Src analysis via Western blot. **e** HMECs were treated with combinations of strain, VEGF (25 ng/mL), SU5416 (3 µM), and a vehicle control for 5 or 15 min. Samples were analyzed through Western blot and stained for total Src. **f**, **g** Quantification of Western blots for total Src in HUVECs with same treatment groups outlined above. Western blots were normalized to β-actin. For **d** and **f**, + *p* < 0.05 versus 0 min NT, * *p* < 0.05 versus NT at same time point, ^ *p* < 0.05 versus V at same time point, # *p* < 0.05 versus ε at same time point. For **e** and **g**, + *p* < 0.05 versus SU5416 with same treatment, * *p* < 0.05 compared to NT at same time point, ^ *p* < 0.05 versus V at same time point, # *p* < 0.05 compared to ε at same time point. Data shown as average + SEM, *n* = 6 for all samples except for **c**, where *n* = 4 excluding SU + mag where *n* = 2. Groups **b**–**g** were compared with Kruskal–Wallis test, followed by post hoc Dunn’s tests. Group **a** was compared with ANOVA, followed by post hoc Tukey HSD tests

The effects of VEGFR-2 inhibition on Src expression were tested in both HMECs and HUVECs. In HMECs, the combination of both exogeneous VEGF and SU5416 inhibitor for 72 h is sufficient to cause a significant decrease in Src of ~ 25% (Fig. 3a, Additional File 1: Fig. S4). However, HUVECs do not show any change between sample groups (Fig. 3b). To further analyze these effects in a more physiologically relevant model, HUVECs were cultured in 3D fibrin gels along with magnetic beads, stimulated by an external magnetic field on an orbital shaker, which generated mechanical stimulation in the absence of stromal cells or secreted factors [43]. The magnetic bead platform is designed to create a mechanically active TME model, where there are deformations or distortions in the matrix around embedded cells; these movements are generated through the movement of the magnetic bead in response to external magnetic stimulation. HUVECs within this system were treated for 7 days with VEGF, a vehicle control, or SU5416; the rings were then digested with nattokinase and analyzed for total Src via Western blot. There are no significant differences due to mechanical stimulation for samples treated with VEGF or vehicle controls; however, SU5416 without a magnet causes a significant increase in Src compared to the vehicle control (Fig. 3c). Furthermore, when magnetic stimulation is applied, there is a smaller effect, suggesting that strain may cause a decrease in Src when HUVECs are treated with a VEGFR-2 inhibitor.

Flexcell plates were also used to analyze rapid, dynamic changes in Src protein levels as a result of uniaxial strain. Similar to previous experiments, HMECs and HUVECs were treated with strain and/or VEGF for 5 or 15 min. Though HMECs show no significant changes, a small increase in Src is seen for samples treated with strain (Fig. 3d). Furthermore, when HMECs were treated with a combination of VEGF, strain, and SU5416, only the 5 and 15 min groups with VEGF display altered Src as a result of VEGFR-2 inhibition (Fig. 3e). HUVEC studies demonstrate a ~ 50% increase in total Src with the addition of strain compared to VEGF only-treated samples, which mimics trends in the HMEC samples (Fig. 3f). When HUVECs were treated with SU5416 and strain, there are no changes in Src levels, as opposed to groups that received only VEGF (Fig. 3g). These studies indicate that strain alone may be sufficient to block Src inhibition in ECs treated with anti-angiogenic drugs.

### Strain in 3D fibrin gels alters vasculogenesis

**Fig. 4:**
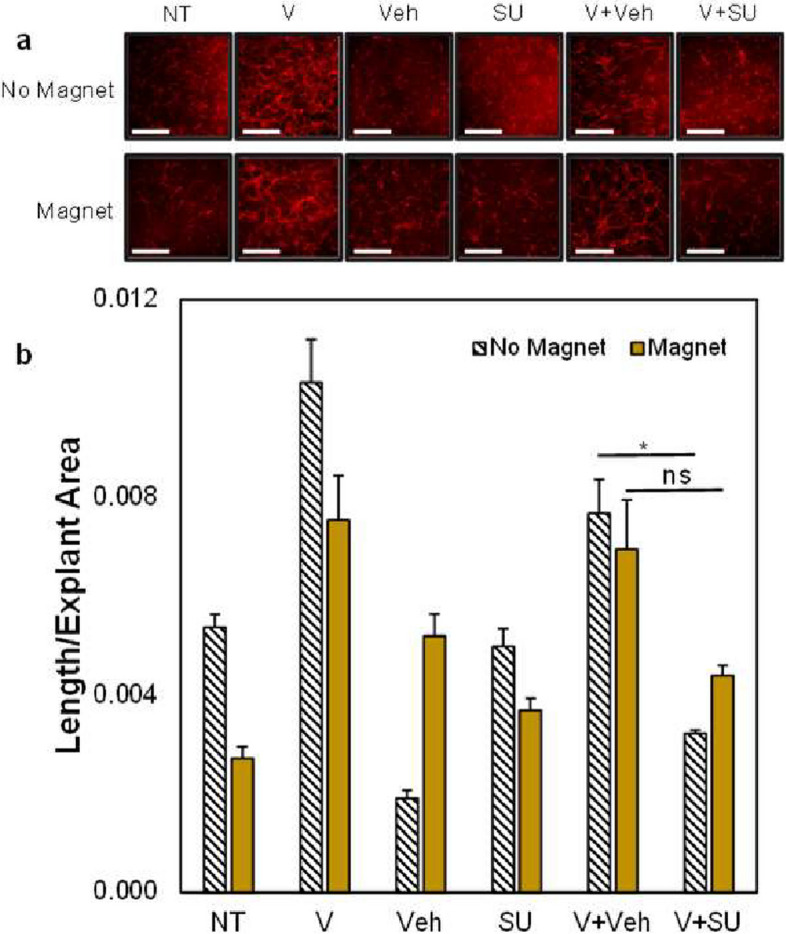
Strain promotes vascular growth even with VEGFR-2 inhibition. **a** HUVECs were grown in fibrin gels with magnetic beads and treated with either no treatment (NT) control media, 25 ng/mL VEGF (V), vehicle control (Veh), and/or 3 µM SU5416 (SU). Samples were either cultured without external magnetic stimulation (No Magnet) or over a rotating magnetic field (Magnet). Gels were stained for VE-cadherin, and representative images are shown. Scale bar = 500 µm. **b** Vessel growth was quantified using AngioTool, and total vessel length was normalized to explant or imaged area. * *p* < 0.05 versus No Magnet of same treatment group. Data shown as average + SEM, *n* = 3 samples. Samples were compared with ANOVA, followed by post hoc Tukey HSD tests

HUVECs were grown in 3D microtissue models made of fibrin gels with magnetic beads with or without external stimulation, with some samples being treated with VEGF and/or SU5416, before quantifying vascular growth in the 3D models. Results demonstrate that, as expected, VEGF promotes blood vessel growth regardless of strain stimulation, but the presence of SU5416 partially inhibits this effect (Fig. 4a, b, Additional File 1: Fig. S4). Still, vessel growth is somewhat restored through mechanical strain generated by the magnetic bead system (Fig. 4b). For samples cultured with mechanical stimulation, there is no difference in vascularization between VEGF plus vehicle groups compared to VEGF plus SU5416 groups; however, removing the magnetic field from these samples does cause a significant decrease when samples are treated with SU5416 (Fig. 4b). This suggests that mechanical simulation plus exogeneous VEGF drives vasculogenesis, even in the presence of anti-VEGFR-2 agents.

### Strain in microfluidic devices promotes angiogenesis

**Fig. 5:**
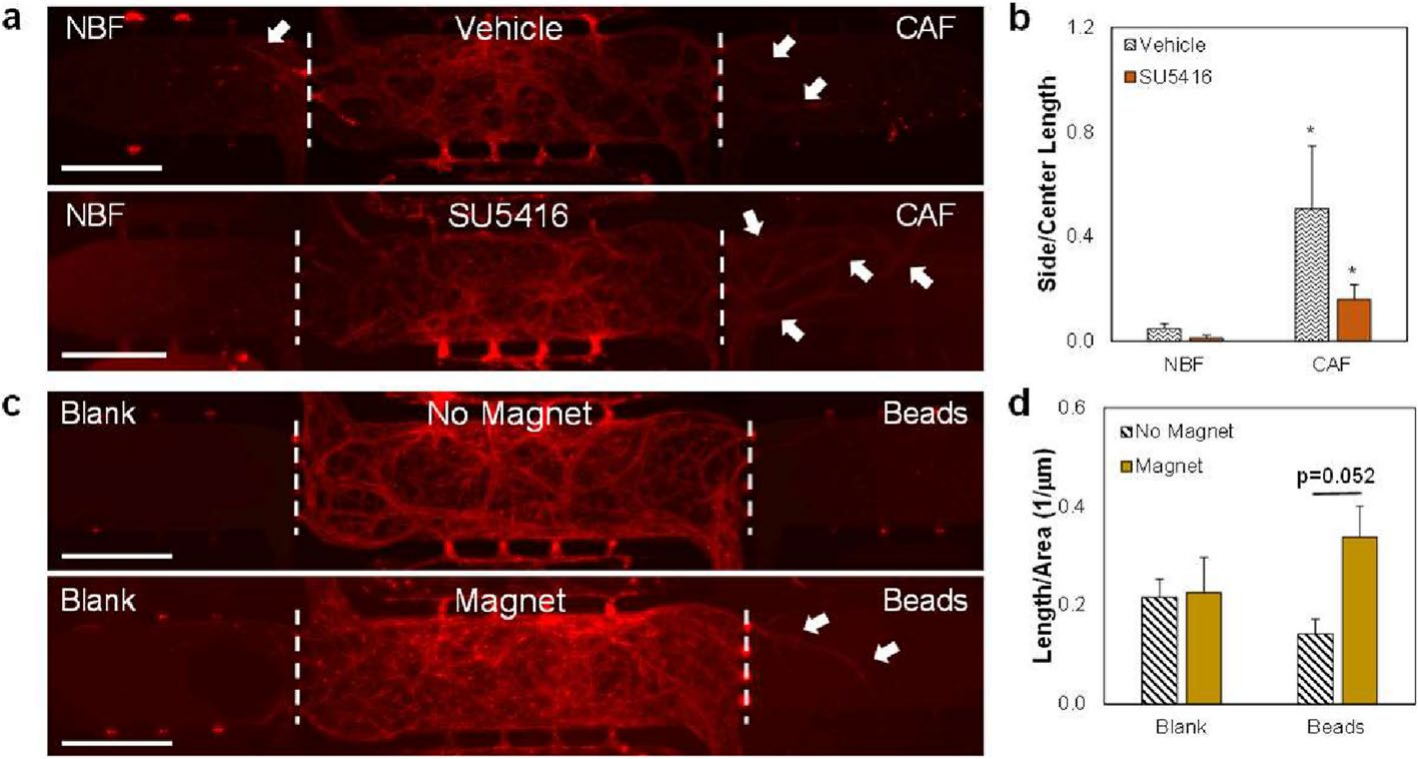
Angiogenesis promoted by mechanical strain in microfluidic devices even without stromal cells. **a** Representative images of multi-chambered microtissue devices with HUVECs and NHLFs in the center chamber with CAFs or NBFs in the side chambers. Devices were grown for 8 days, with treatments being added after 4 days. Devices were stained for VE-Cadherin (red). White arrows identify vessel structures. Dashed lines show interfaces between chambers. Scale bar = 500 µm. **b** Quantification of vessel growth in the side chambers using AngioTool and FIJI, shown as total length in side chamber normalized to total length in center chamber for each microtissue model. * *p* < 0.05 versus Vehicle-treated NBF. **c** Representative images of multi-chamber microtissue devices with HUVECs and NHLFs in the center chamber and either blank (cell- and bead-free) fibrin only or fibrin gels plus magnetic microbeads in the side chambers. Samples received either no magnetic stimulation (No Magnet) or were cultured above a rotating magnetic field (Magnet). Systems were stained for VE-Cadherin (red) after 7 days of culture time. White arrows identify vessel structures. Dashed lines show interfaces between chambers. Scale bar = 500 µm. **d** Quantified results of vessel growth in side chambers; vessel in side chambers was first normalized to explant or gel area for the chamber, then all values were normalized to the total length/explant area of the side chamber. * *p* < 0.05 versus No Magnet. For **a**, **b**, *n* ≥ 7 devices per condition. For **c**, **d**, *n* = 4 for Blank + No Magnet, *n* = 5 for Blank + Magnet, and *n* ≥ 7 for both Beads groups. Data shown as average + SEM, *n* ≥ 3 samples. Group **b** was compared with Kruskal–Wallis test, followed by post hoc Dunn’s tests. Group **d** was compared with ANOVA, followed by post hoc Tukey HSD tests

A microfluidic device was used to study strain-induced angiogenesis with and without VEGFR-2 inhibition [48]. Systems were loaded with HUVECs and NHLFs in the center chamber to generate a lumenized vascular network from which sprouting angiogenesis could be measured into side chambers (Fig. 5a). CAFs loaded into one of the side chambers represent the mechanically active TME, through the increased contractile behaviors that induce matrix distortions, while NBFs represent normal tissue. Devices were set up so that interstitial flow exited the center chamber and through the side chambers, effectively “washing out” secreted factors from both CAFs and NBFs away from the angiogenic front and thereby focusing the studies on only mechanical interactions at the interfaces between chambers. Results show that chambers containing CAFs induce significantly higher levels of angiogenesis compared to chambers with NBFs, in line with our previous work, and this occurs even in the presence of SU5416 (Fig. 5b) [48]. To verify that soluble factors do not have an impact on angiogenesis, additional devices were made with HUVECs and NHLFs in the center chamber, fibrin only (cell-free) gels in one side chambers with magnetic beads in the opposite side chamber. Results show that when beads were stimulated by a magnet, there is a small increase (*p* = 0.052) in the length of vessels that grow into mechanically stimulated chamber versus chambers that did not receive magnetic stimulation, suggesting that strain may be sufficient to increase angiogenic activity (Fig. 5c, d).

## Discussion

### Alterations in VEGFR-2 phosphorylation due to mechanical strain

To determine if biomechanical forces could alter VEGFR-2 activation levels, we designed studies using multiple EC lines as well as both biochemical and biomechanical stimuli before examining protein levels of phosphorylated residues of VEGFR-2. Previous work has shown that alterations in the mechanical environment can affect VEGFR-2 phosphorylation; Chen et al. notably demonstrated that attaching VEGF to the ECM resulted in prolonged Y1214 phosphorylation compared to soluble VEGF [24]. We wanted to investigate how mechanical strain could also affect VEGFR-2 phosphorylation. In 2D without any strain, both HMECs and HUVECs treated with VEGF-free media displayed relatively low VEGFR-2 phosphorylation, particularly for Y1214 (Fig. 2). However, both cell lines on Flexcell plates show somewhat increased phosphorylation at Y1214 at 5 min in samples treated with strain compared to samples without strain (Fig. 2c, i). When compared to 0 min (no treatment) phosphorylation levels, pY1214 levels appeared sustained and prolonged in HMECs in samples treated with strain for up to 15 min (Fig. 2i). The addition of VEGF further increased this Y1214 phosphorylation. The combination of strain and VEGF may more accurately reflect the in vivo TME, where increases in VEGF are observed during tumor progression alongside altered and enhanced mechanical forces [40]. The strain placed on the ECs mimics local strains produced by CAFs as previously studied through a bead displacement assay, which further suggests that these changes in VEGFR-2 phosphorylation patterns may appear within the in vivo TME [43]. We did not determine the minimum or maximum strain percentages required to produce an alteration in VEGFR-2 activity; however, NBFs, which cause smaller magnitude matrix deformations than CAFs, do not promote as much blood vessel growth as the more mechanically active CAFs [43]. While a full parameterization sweep of elongation magnitudes and strain frequencies was beyond the scope of this study, such studies could be useful in the future for determining the specific mechanism by which strains promote conformation changes or regulate transphosphorylation events in VEGFR-2 that may play a role in the mechanoactivation of this receptor. Regarding Y1054/Y1059 residues, HMECs showed no significant increase in phosphorylation caused by strain; however, HUVECs presented with increased phosphorylation at this residue site when treated with strain with or without VEGF for 15 min (Fig. 2b, h). Total VEGFR-2 levels did decrease with the addition of VEGF at both time points, likely due to activation and subsequent internalization and degradation (Fig. 2a, g) [34]. Our data suggest that external strains can lead to prolonged activation of the VEGFR-2 receptor at both the Y1054/Y1059 and Y1214 residues, depending on cell type, which may be at least partially independent of ligand-based signaling.

Furthermore, results demonstrated that both Y1054/Y1059 and Y1214 are mechanically responsive phosphorylation sites, suggesting a novel mechanoactivation of the receptor through tensile strains. Previous studies have primarily focused on VEGFR-2 mechanosensing through shear stress or substrate stiffnesses [51–53]. However, the Y1054/Y1059 sites are necessary for full kinase activity, and other phosphorylation events on VEGFR-2 may impact phosphorylation rates at this residue [20]. A full investigation of this is beyond the scope of the current study, but this fact suggests that additional research is needed to fully define mechanoactivation of VEGFR-2. Regarding Y1214, this residue became phosphorylated due to the biochemical stimulation of VEGF, showing that activation of VEGFR-2 through this site is likely a combination of mechanical and chemical cues. In a 2D environment without strain, HUVECs and HMECs that were treated with SU5416 for 3 days showed a decrease in Y1054/Y1059 and Y1214 phosphorylation, even with the addition of VEGF; however, this pattern is altered with the addition of strain (Fig. 1b, d). Interestingly, the addition of strain and VEGF showed increased pY1054/Y1059 without the need of exogenous VEGF at both 5 and 15 min, depending on cell type (Fig. 2e, k). Phosphorylation of Y1214 follows a similar pattern when samples were treated with the vehicle control; however, phosphorylation decreases with the addition of SU5416 (Fig. 2f, l). These changes in tyrosine phosphorylation indicate that anti-angiogenic therapies in a mechanically active TME are unlikely to completely stop blood vessel growth due to the increased VEGFR-2 activity initiated by strain. Such strains may exist in the TME due to a variety of factors, including factors such as increased deposition and remodeling of the ECM as well as high levels of contractility of CAFs [43, 54]. This mechanical strain, along with VEGF produced by tumor cells and CAFs, may be necessary to cause sustained VEGFR-2 phosphorylation in the presence of SU5416, which is a significant area of interest to develop effective anti-angiogenic cancer therapies.

### Increased mechanoactivation of VEGFR-2 downstream factors

While VEGFR-2 is upstream of many prominent cell signaling pathways, our studies focused on the mechanotransducer Src, which is linked to increased permeability of vascular structures in ECs exposed to VEGF [30, 55]. The increased permeability is a crucial first step in angiogenesis, further supporting Src as a key potential downstream factor of mechanically activated VEGFR-2 [56, 57]. HUVECs cultured in 2D treated with VEGF, SU5416, or a combination of both showed no significant difference in Src expression (Fig. 3b). This may be due to compensatory mechanisms outside of VEGFR-2 regulating Src expression; however, these data were collected after 72 h of culture time and may not have captured rapid changes to Src regulation in HUVECs. This is partially supported by results from our strain studies, where HUVECs treated with mechanical strain for 15 min displayed an increase in Src compared to samples treated with VEGF (Fig. 3f). Previous literature has shown that activated Src is more unstable than inactive Src; therefore, active Src may be more quickly degraded [58]. Thus, the observed increase of Src protein levels in VEGF-treated cells could be due to Src inactivity and higher stability. Results from studies with the immortalized EC line HMECs suggested a similar trend compared to HUVECs; however, HMECs may present more drastic changes in Src expression due to both VEGF and SU5416 treatments (Fig. 3a, d). For both ECs treated with combinations of strain and SU5416, strain appeared to block the effect of SU5416 compared to the VEGF treatment, which generally lowered Src expression (Fig. 3e, g). This suggests that strain may cause a compensatory mechanism to rescue ECs from the effects of anti-angiogenic therapies, which likely applies to a wide variety of pathways. VEGF alone is insufficient to combat these drugs, but strain alters Src signaling even in the presence of VEGFR-2 inhibitors. Interestingly, HUVECs grown in a 3D fibrin gel exhibited an increase in Src in both mechanically active and -inactive environments when treated with SU5416. The higher levels of Src observed in mechanically inactive samples (no magnet stimulation) may be due to a buildup of inactive Src (Fig. 3c). The addition of mechanical strain through magnetic beads and a rotating magnetic force caused a downward trend in Src levels in samples treated with SU5416, potentially indicating that Src was being degraded after activation. Also, other VEGFR-2 phosphorylation sites such as Y1175 are known to be mechanically responsive to shear stresses, with Src being linked to Y1175 phosphorylation [59, 60]. Additional studies of those residues along with Src activation could elucidate how VEGFR-2 acts as a mechanoreceptor that mediates tensile strains to activate Src in the TME.

### Angiogenesis upregulation promoted by VEGFR-2 as a mechanoreceptor

While we know that VEGFR-2 is a critical driver of angiogenesis in the TME, previous studies did not consider how biomechanical strain drives VEGFR-2 regulated vessel growth. To investigate whether mechanical strain actually promotes the development of blood vessels, we used magnetic beads to create mechanically active or inactive (control) environments. In fibrin gels, VEGF increased vasculature length and, as expected, VEGFR-2 inhibition via SU5416 limited vessel growth (Fig. 4b). In ECs that were not strained, VEGF plus SU5416 caused ~ 50% decrease in vascular growth versus VEGF plus Veh groups; however, strained cells did not show a significant decrease in vascular development, suggesting that mechanical strain partially restored the angiogenic properties of ECs treated with a VEGFR-2 inhibitor. These results support the idea that increased mechanoactivation of VEGFR-2 occurs even in the presence of inhibitors. Though many anti-angiogenic therapies attempt to inhibit VEGFR-2, both heightened levels of secreted VEGF and mechanical activity are still present in the environment, which may allow for continued blood vessel growth and limit treatment efficacy. Our studies utilized a non-flow system that undergoes vasculogenesis to form blood vessels; vasculogenesis can occur in the TME as well [5, 61]. Therefore, we wanted to continue in a more specific model to investigate mechanically stimulated angiogenesis from existing vascular networks. Microtissue models with three adjacent tissue regions in series were treated with an anti-angiogenic treatment and showed increased angiogenesis in the mechanically active side chambers containing CAFs compared to NBFs, which are less mechanically active (Fig. 5b). This model was previously developed and used to describe mechanical activity of the fibroblasts in terms of matrix displacements or distortions generated through contractility events [48]. The media feeding regime was set up with “outward flow,” so that media flowed from the center chamber into the side chambers (Fig. 5), thus ensuring that soluble factors produced by stromal cells within the side chambers are flushed away from the center chamber interface. Therefore, these studies focus on the mechanical interaction of CAFs or NBFs with the cells in the center chamber, to show that such mechanical strains drive angiogenic growth while isolating biomechanical stimulation from biochemical stimulation due to secreted factors.

To further support the conclusion that mechanical forces are sufficient to drive angiogenesis without paracrine secreted growth factors, we used the magnetic bead system with a rotating magnet to generate strains that mimic what CAFs produce in 3D microtissue TME models but without stromal secreted factors present. These studies further support that mechanical forces alone, generated by the magnetic bead system, appeared to enhance angiogenesis in environments where no stromal cells are present (Fig. 5d). In other words, angiogenesis specifically occurred into the chambers containing a mechanically active TME model. Furthermore, this is significant because in the experimental setup, blood vessels were growing with the direction of flow (from center chamber outward to side chambers), which is different from normal angiogenic behavior where vessels grow against the interstitial flow gradient, possibly through integrin signaling [62, 63]. This suggests that mechanical strains may promote angiogenesis through a different mechanism. Due to altered biomechanical stimuli present in the TME, blood vessels may continue to grow and provide tumors with enough nutrients to progress even in the presence of anti-angiogenic medicine.

In context of the TME, mechanically active CAFs, a stiffer ECM, and higher interstitial fluid pressure can cause strain on ECs that promotes enhanced angiogenesis and tumor progression. Furthermore, CAFs secrete large amounts of VEGF, along with other growth factors. Both chemical and mechanical stimuli may cause increased VEGFR-2 activation and upregulation of angiogenesis. VEGFR-2 inhibitors such as SU5416 may prove ineffective when both biomechanical and biochemical signals are combined in the TME, resulting in vessel growth even in the presence of anti-angiogenic therapies.

## Conclusions

Inhibiting angiogenesis within the TME should prevent tumor growth and progression; however, current anti-angiogenic therapies have proven to be insufficient by themselves and even sometimes when combined with other chemotherapies [29]. We hypothesize that increased strain present in the TME may cause mechanoactivation of VEGFR-2 even in the presence of angiogenic inhibitors. In this paper, we demonstrated that strain and strain plus VEGF can cause increased VEGFR-2 phosphorylation in ECs, along with a potential promotion of Src activity. Still, some differences between HMECs and HUVECs are apparent, suggesting the need for further study of these cell types with additional VEGFR-2 tyrosine residues. Furthermore, angiogenesis was shown to be upregulated in 3D mechanically active environments caused by CAFs in the TME even when treated with SU5416 to inhibit VEGFR-2. Due to these results, we conclude that VEGFR-2 acts as a mechanoreceptor to tensile strains, and its activation is upregulated due to both biochemical and biomechanical cues present within tumors. In order to fully understand how to inhibit angiogenesis and tumor progression, further research is necessary to comprehend the complete mechanism of VEGFR-2 signaling.

## Methods

### Cell culture

Human umbilical vein endothelial cells (HUVECs) (Lonza, C2517A) were grown in EGM-2 (Lonza, CC-3162) and used between passages 2–6. Human microvascular endothelial cells (HMECs) (HMEC-1, ATCC CRL-3243) were grown in MCDB 131 (Gibco, 103720198) supplemented with 2 ng/mL EGF (Fisher Scientific, 50–813-058), 1 µg/mL hydrocortisone (Sigma-Aldrich, H0888), 10% HI FBS (Gibco, 10–082-147), and 10 mM L-glutamine (Gibco, 25–030-081). All ECs were grown on tissue culture plates coated with 1% gelatin (Sigma-Aldrich, G1890) in DPBS (Gibco, 14190–250). Using both a primary cell line (HUVECs) and an immortalized cell line (HMECs) provided flexibility in optimizing and performing experiments. Immortalized cancer-associated fibroblasts (CAFs) from a breast cancer patient were previously developed, along with patient matched immortalized normal breast fibroblasts (NBFs) [64]. The fibroblasts were grown in DMEM (Gibco, 11995065) with 2 mM L-glutamine, 1% sodium pyruvate (Gibco, 11360070), 1% non-essential amino acid solutions (Gibco, 1140–050), 100 U/mL penicillin streptomycin (Fisher Scientific, 15–140-122), and 10% non-HI FBS (Gibco, 26140079). Normal human lung fibroblasts (NHLFs) (Lonza, CC-2512) were cultured in the same media as other fibroblasts and used between passage numbers 2–8. For cell harvesting, 0.25% trypsin (Gibco, 25–200-072) was used. All cells were cultured at 37 °C and 5% CO_2_. To stimulate angiogenic signaling, exogenous VEGF (Peprotech, 100–20) was added to cell cultures at 25 ng/mL for a range of timepoints A VEGFR-2 small molecule inhibitor, SU5416 (abbreviated as SU) (Sigma-Aldrich, S8442) was dissolved in DMSO (Sigma-Aldrich, D8418) and used at 3 µM in cell media; DMSO was used as vehicle control (abbreviated as veh) in inhibitor studies. The inhibitor SU5416 causes this by blocking VEGFR-2 tyrosine kinase activity by binding to the intracellular domain of VEGFR-2 to interfere with phosphorylation [65–67]. Finally, no treatment (abbreviated as NT) samples were included as controls. In strain studies, 0 min NT groups were lysed after being plated 24–48 h previously in EGM-2 (2 ng/mL VEGF) without subsequent media changes. In these experiments, 5 and 15 min NT groups were treated with 0 ng/mL VEGF before being lysed.

### Magnetic bead preparation

Iron oxide beads with a diameter of 5 µm and a tosyl-group surface coating (Dynabeads, M-450 ThermoFisher, 14013) were coated in thrombin to be incorporated into fibrin gels in a previously described protocol [68]. This protocol permits mechanical stimulation of ECs without stromal cells and acts as a control to induce mechanical stimulation but eliminate stromal secreted factors. The magnetic beads are effectively, but not chemically, crosslinked in the fibrin gels and external magnetic stimulation causes matrix deformations similar to CAFs or other types of stromal cells. To coat the beads, 100 µl of Dynabead stock solution was washed with DPBS then resuspend in 100 µl sterile bicarb buffer (Sigma-Aldrich, C3041) and 100 µl of 50U/mL thrombin (Sigma-Aldrich, T4648) solubilized in 0.1% BSA (Sigma-Aldrich, A2153) in DPBS. The mixture was incubated on a tube rotator overnight at 4 °C then washed twice in 0.1% BSA in DPBS before resuspension in 200µL 0.1% BSA in DPBS. Mechanical stimulation is achieved by culturing the 3D microtissues over a rotating magnetic field on an orbital shaker; the control studies include magnetic beads but are not cultured over the magnet.

### 3D fibrin ring assay

A non-flow 3D TME microtissue model was previously developed in our lab where a 10 mg/mL fibrin disk is embedded with cells [43]. In order to form a 3D fibrin gel, 1-cm rings (ID = 0.8 cm) made of polydimethylsiloxane (PDMS) (Dow Corning, Sylgard 184) were placed on glass coverslips (Fisher, 22–293232). These were then autoclaved and placed in a 24-well plate for cell culture studies. Fibrinogen (Sigma-Aldrich, F8630) was solubilized in DPBS without calcium or magnesium ions. Cells were resuspended in this fibrinogen to create a final concentration of 1 × 10^5^ cells/gel with a minimum of three replicates generated for each experimental group. Thrombin was added to a final concentration of 3U/mL before the plates were incubated at 37 °C for 30 min to allow for full fibrin polymerization. The microtissues were then fed with 1 mL EGM-2, with fresh media added every 2 days. In some studies, samples were treated with 25 ng/mL VEGF, DMSO, 3 µM SU5416, and combinations of VEGF/DMSO or VEGF/SU5416. After 7 days, the rings were either fixed for immunofluorescence vessel growth analysis or digested with nattokinase for protein analysis.

### Microfluidic device studies

Our lab has previously designed a multi-microtissue microfluidic platform for biomechanical investigations of angiogenesis [48]. The design involves three microtissue tissue regions in series, herein referred to as the left, center, and right chambers, with fluid lines on the top and bottom of each chamber that allow for control over flow direction between regions. A 10 mg/mL fibrin gel can be injected into each of the chambers, with different cell populations and concentrations depending on experimental setups. For angiogenic studies, HUVECs and NHLFs were seeded into the center chambers at 1.0 × 10^7^ cells/mL each in a 1:1 ratio; NHLFs acted as stromal cells which are necessary to establish a stable and anastomosed vascular network [49, 69]. Devices were fed with EGM-2, and media was changed daily. Device feeding was performed through “outward flow regime” where media enters the center chamber and flows to the side chambers, effectively preventing any potential soluble factors produced in the side chambers from diffusing into the center [48]. In experiments with stromal cells, CAFs or NBFs were loaded into side chambers of the devices at a concentration of 1.25 × 10^7^ cells/mL. A series of experiments utilized thrombin-coated magnetic beads mixed with the fibrin gel to create a side chamber with matrix distortions without secreted factors [43]. These systems were either cultured above a magnet on an orbital shaker, to provide mechanical stimulation and induce matrix distortions, or in a magnet-free (no magnet) environment as a control. Blank fibrin gels were used in the opposing side chambers of these devices as a cell-free control. Systems were cultured for 7–8 days at 37 °C and 5% CO_2_. Only devices used in SU5416 studies were cultured for 8 days, with the treatment (Veh or SU5416) being added for the last 4 days. All other device studies were cultured for 7 days before fixation.

### Immunofluorescence staining and imaging

For image analysis of vasculogenesis angiogenic vessel growth, 3D in vitro TME models were fixed and stained vascular endothelial cadherin (VE-Cad) based on previously developed protocols [43, 48]. Briefly, fibrin ring samples were washed with 1 × PBS (Fisher Scientific, BP39920) then fixed using 10% formalin (Fisher Scientific, SF100-4) for 20 min at room temperature. Rings were then washed and blocked in Abdil (PBS plus 2% BSA and 0.1% Tween-20 [Fisher Scientific, AAJ20605AP]) for 1 h. Primary antibodies were diluted in Abdil (VE-Cad 1:500; abcam, ab33168) and samples were incubated overnight on a rotator plate at 4 °C. The next day, samples were washed with PBS + 0.1% Tween-20, 4 times for 20 min each. Secondary antibody Alexa Fluor 555 (ThermoFisher, A31570) was diluted in Abdil at 1:500 before incubation overnight at 4 °C on a rotator plate. Samples were again washed 4 × for 20 min each with PBS + 0.1% Tween-20 before imaging. Device fixation followed a similar protocol, except all incubation times were 48 h for each step to allow for diffusion throughout the microtissues. Samples were imaged at 10 × using an inverted epifluorescence Olympus Microscope (IX83), capturing 100 µm Z-stack with a step size of 2 µm for each sample. Images were stitched together to create a full-length picture for each device [70]. Images were processed in FIJI to generate Max-Z projections, and vascular growth was quantified using AngioTool [71]. For vasculogenic ring samples, total vessel length was normalized to the gel area for each image. For microfluidic devices, vessel growth in side chambers was either normalized to total vessel length in the center chamber or was first normalized to the gel area imaged in each side chamber, then normalized to total length in the center chamber.

### Flexcell studies

A commercially available Flexcell system (Flexcell, FX-6000 T) was used to strain HMECs and HUVECs to study changes in protein expression levels in the VEGFR-2 pathway. Briefly, cells were cultured at 5 × 10^5^ per well on collagen-I coated uniaxial Flexcell plates for 24–48 h before being moved to the baseplate for tensile strain studies. HMECs and HUVECs were exposed to 0 ng/mL or 25 ng/mL VEGF and oscillatory strains at 9% elongation at 0.3 Hz for 0, 5, and 15 min. Elongation accuracy was determined through previous calibration studies performed by Flexcell. The strain magnitude was chosen as it mimics strains generated by the CAFs from our previous studies, while the rate reflects normal human respiration rates [43]; therefore, our strain regime is a highly physiologically relevant set of parameters. Samples were lysed in RIPA buffer made with 50 mM Tris 7.4, 150 mM NaCl (Sigma-Aldrich, S3014), 0.25% sodium deoxycholate (Sigma-Aldrich, L3771), 1% Triton X-100 (Sigma-Aldrich, T8787), 1 mM EDTA (Sigma-Aldrich, E9884), and 5 mM sodium fluoride (Sigma-Aldrich, S6776) plus 1:100 HALT protease and phosphatase inhibitor (ThermoScientific, 78,441). All samples were run in triplicate. Each study also had a control Flexcell plate that did not receive strain.

### Nattokinase

To digest fibrin gels and collect cells for protein analysis, we utilized a nattokinase solution based on previously published protocols [72]. Briefly, nattokinase (MedChem Express, HY-P2373) was solubilized 1 mM EDTA in 1 × PBS, resulting in a final concentration of 100 fibrin-degrading units (FU)/mL. Fibrin gels containing HUVECs and thrombin-coated magnetic beads were mixed with 150 µl nattokinase solution for every 3 rings and incubated at 37 °C for 1 h or until gels were dissolved. For these studies, 9 rings per condition were generated; to enhance protein collection for Western blots, 3 rings were pooled during the digestion protocol. Cells were then spun down and washed in DPBS, then spun down again and resuspended in 50–75 µl RIPA plus 1:100 HALT.

### Western blot

Standard Western blot protocols were followed. Protein concentrations were analyzed via Bradford assay and 50 µg of protein was loaded for each sample. Lysates were run on 8% SDS-PAGE gels, then proteins were transferred onto PDVF membranes. Membranes were blocked in 5% milk in TBST or 5% BSA in TBST for phosphorylated targets. Blots were stained for VEGFR-2 (1:1000; Cell Signaling Technology, 2479), pY1054/Y1059 VEGFR-2 (1:1000–1:200; Invitrogen, 44-1047G), pY1214 VEGFR-2 (1:1000–1:200; Invitrogen, 44–1052), Src (1:1000; Cell Signaling Technology, 2109), pY418 Src (1:1000; abcam, ab40660), GAPDH (1:1000; Cell Signaling Technology, 2118), and β-actin (1:40,000; Sigma-Aldrich, A1978); all incubations were overnight at 4 °C on a rotator plate. Secondary antibodies were diluted in 5% milk in TBST or 5% BSA in TBST for phosphorylated targets for 2 h at room temperature before using chemiluminescent detection (ECL, ThermoScientific 32,106 or Femto, ThermoScientific 34,095). Blots were imaged using a GelDoc or Licor system and analyzed via densitometry through FIJI. Phospho antibodies were quantified at mature protein weight, with exception for pY1054/Y1059, which was analyzed for the 160 kDa cleavage product in some instances. Either GAPDH or β-actin were used as a loading control for all studies.

### Statistical analysis

For all studies, data are reported as averages + SEM with a minimum of 3 replicates. Specific replicate numbers are described in the figure legends for each set of data, and individual data points are listed in Additional File 2. To determine statistical significance, first data sets were screened for normality via Shapiro–Wilk tests. If all data sets within an experiment were normal, we performed ANOVA with post hoc Tukey HSD tests as needed; for any experimental data sets that had at least one non-normal sample distribution, we performed Kruskal–Wallis tests followed by post hoc Dunn’s tests. Statistical calculations were completed using the Real Statistics Resource package for Excel (https://real-statistics.com/). Statistical significance was considered at *p* < 0.05.

### Supplementary Information

**Additional file 1: Figures S1-S4. Fig. S1.** labelled Western blots for data in Fig. 1. **Fig. S2.** labelled Western blots for data in Fig. 2. **Fig. S3.** quantification of pY1054/Y1059 and pY1214 contrasting data in Fig. 2e, f, k, l normalized to β-actin as opposed to total VEGFR-2. **Fig. S4.** labelled Western blots for data in Fig. 3.

**Additional file 2.** Raw data points for all figures.

## Data Availability

Images generated and analyzed in this study are either shown in the article or are included in Additional File 1. The file contains full membrane images for Western blot studies shown in the manuscript. Raw data values for all figures are provided in Additional File 2.

## Abbreviations

CAF: Cancer-associated fibroblast
EC: Endothelial cell
ECM: Extracellular matrix
HMEC: Human microvascular endothelial cell
HUVEC: Human umbilical vein endothelial cell
NBF: Normal breast fibroblast
NHLF: Normal human lung fibroblast
TME: Tumor microenvironment
VEGF: Vascular endothelial growth factor
VEGFR-2: Vascular endothelial growth factor receptor-2
