# Supplementary material for: Biomechanical stimulation promotes blood vessel growth despite VEGFR-2 inhibition
Source: BMC Biol. 2023 Dec 10;21:290. doi: 10.1186/s12915-023-01792-y (PMC10712065; doi:10.1186/s12915-023-01792-y)
Supplement: Supplementary file 1 — Additional file 1: Figures S1-S4. Fig. S1. labelled Western blots for data in Fig. 1. Fig. S2. labelled Western blots for data in Fig. 2. Fig. S3. quantification of pY1054/Y1059 and pY1214 contrasting data in Fig. 2e, f, k, l normalized to β-actin as opposed to total VEGFR-2. Fig. S4. labelled Western blots for data in Fig. 3. [file 12915_2023_1792_MOESM1_ESM.pdf]

1a

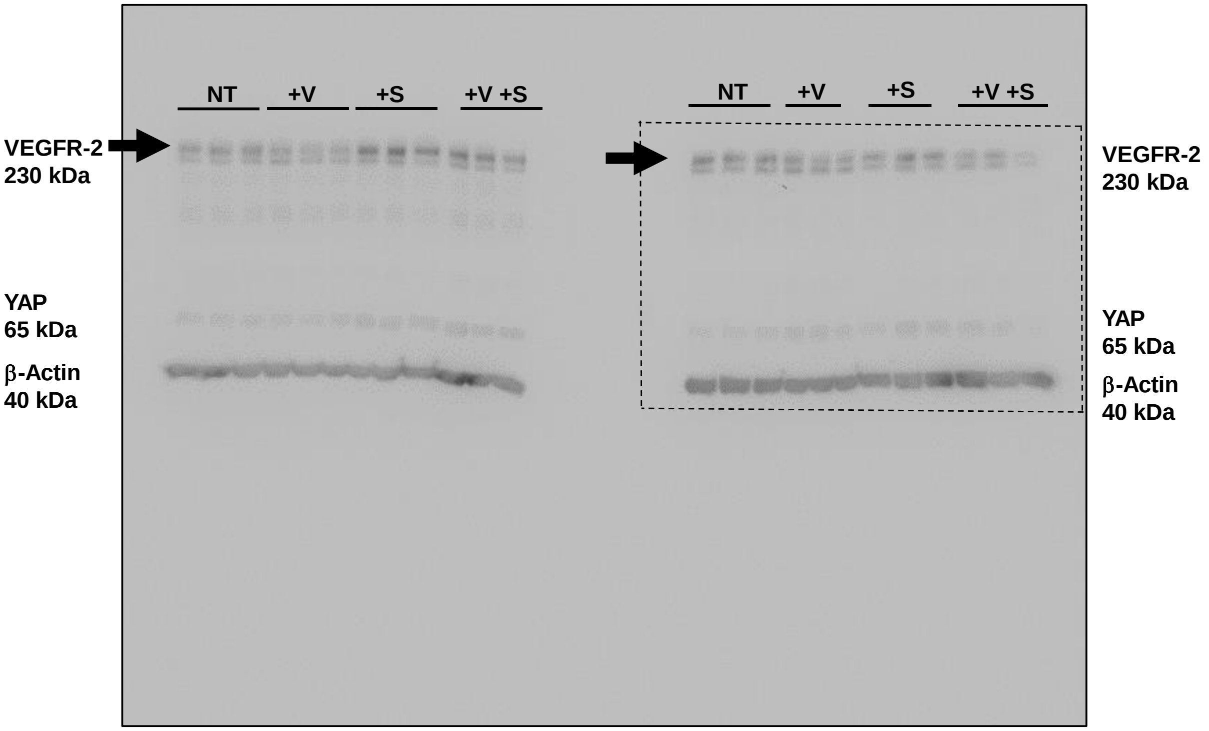
NT
+V
+S
+V +S
VEGFR-2
230 kDa
YAP
65 kDa
β-Actin
40 kDa
NT
+V
+S
+V +S
VEGFR-2
230 kDa
YAP
65 kDa
β-Actin
40 kDa

1b

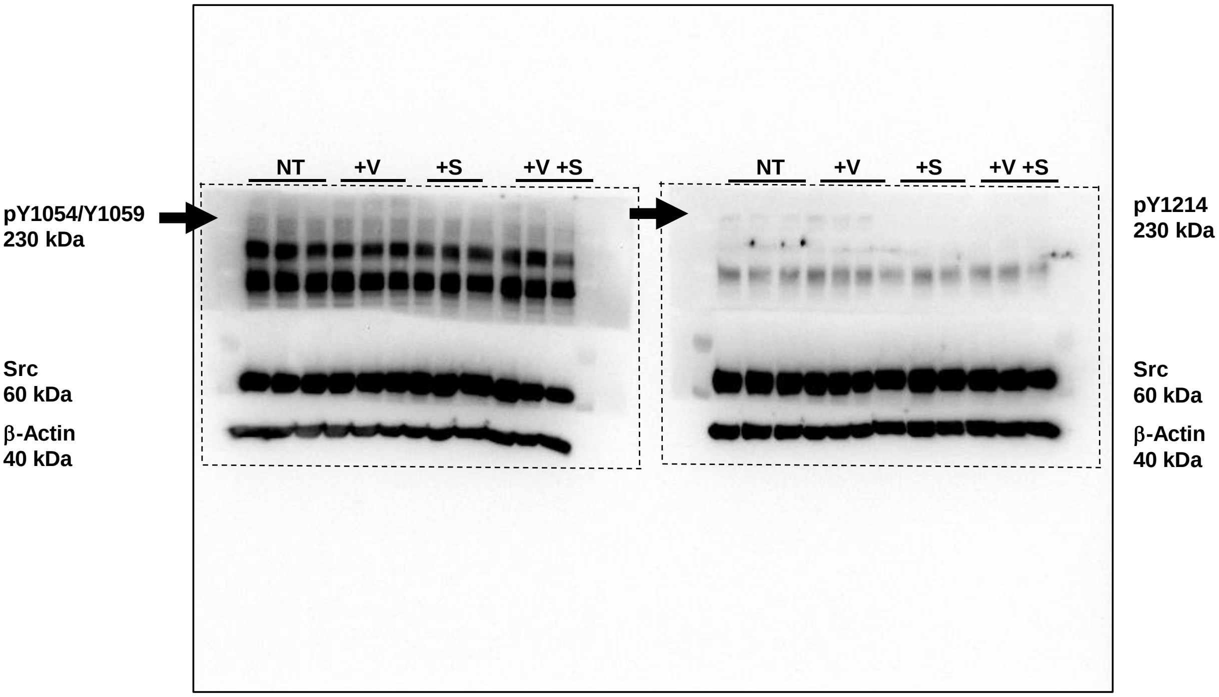
NT
+V
+S
+V +S
pY1054/Y1059
230 kDa
Src
60 kDa
β-Actin
40 kDa
NT
+V
+S
+V +S
pY1214
230 kDa
Src
60 kDa
β-Actin
40 kDa

1c

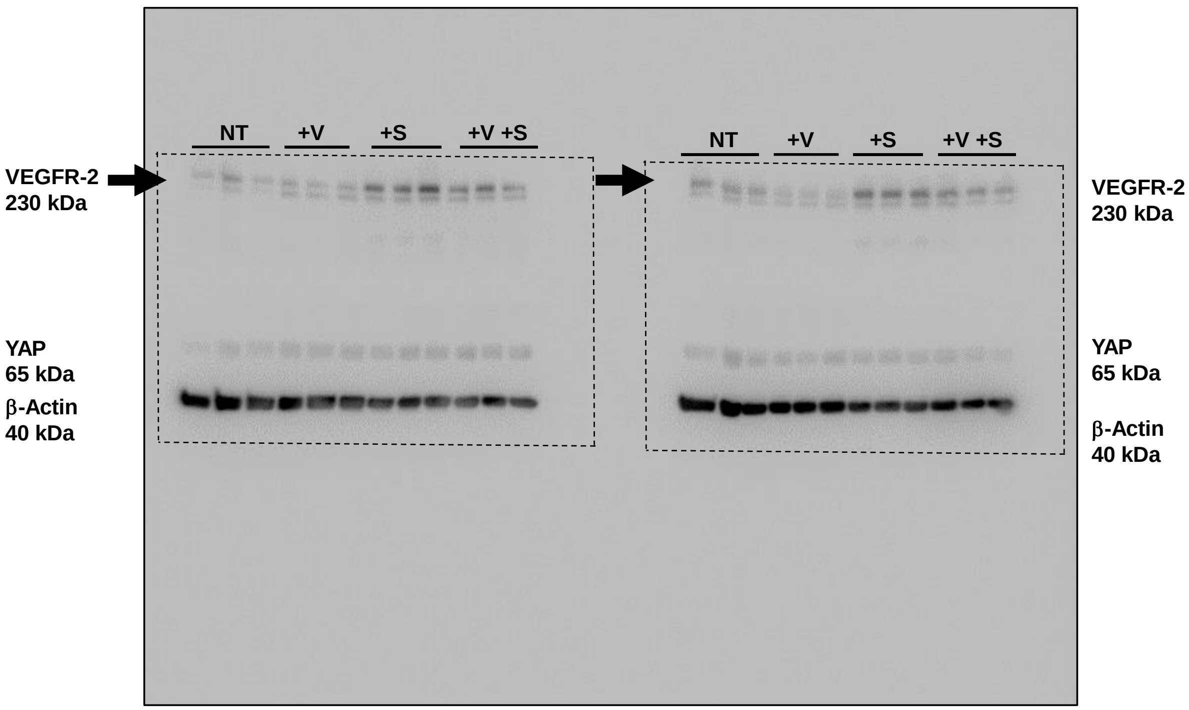

1d

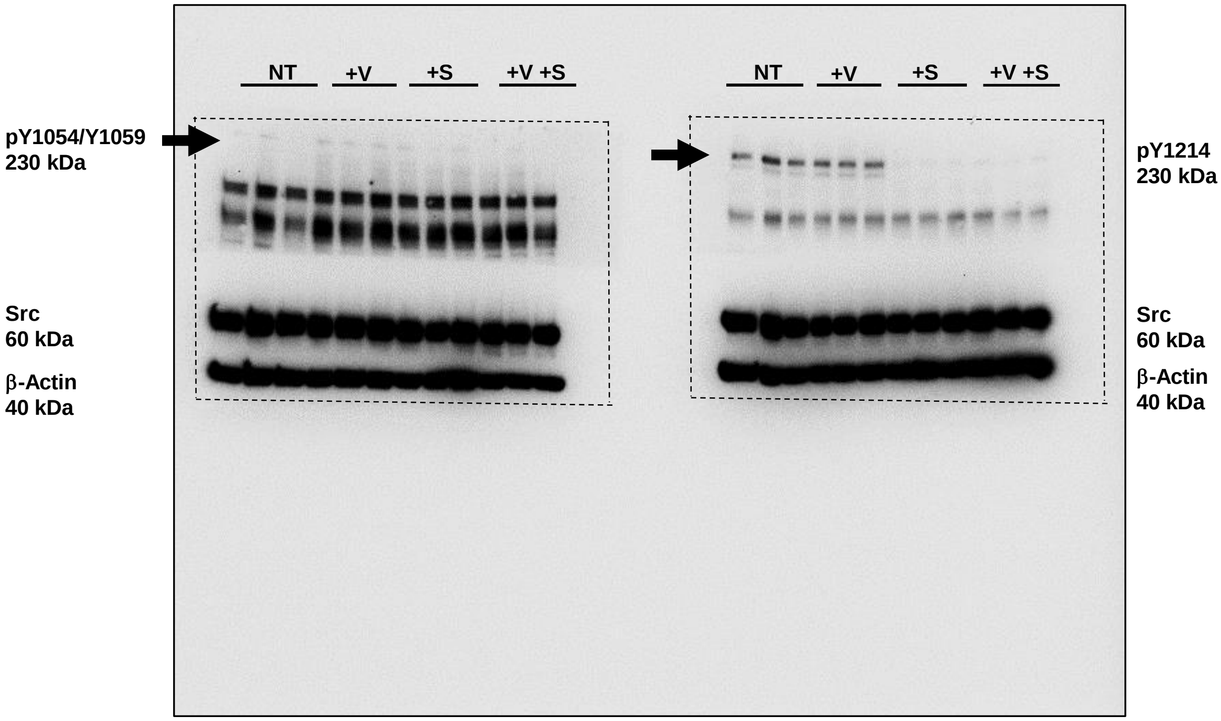

Supplemental Fig. 1 – Labelled Western Blots for Data in Fig. 1. This figure matches Fig. 1 in the main text. **(a)** HMECs were either given no treatment (NT), exogenous VEGF (+V), SU5416 (+S), or both exogenous VEGF and SU5416 (+V +S). Blots were stained for total VEGFR-2, **(b)** pY1054/Y1059, and pY1214. **(c-d)** HUVECs received the same treatments and stains. All blots were

also stained for YAP or Src and β-actin as a loading control. Arrows indicate VEGFR-2 bands quantified.

2a

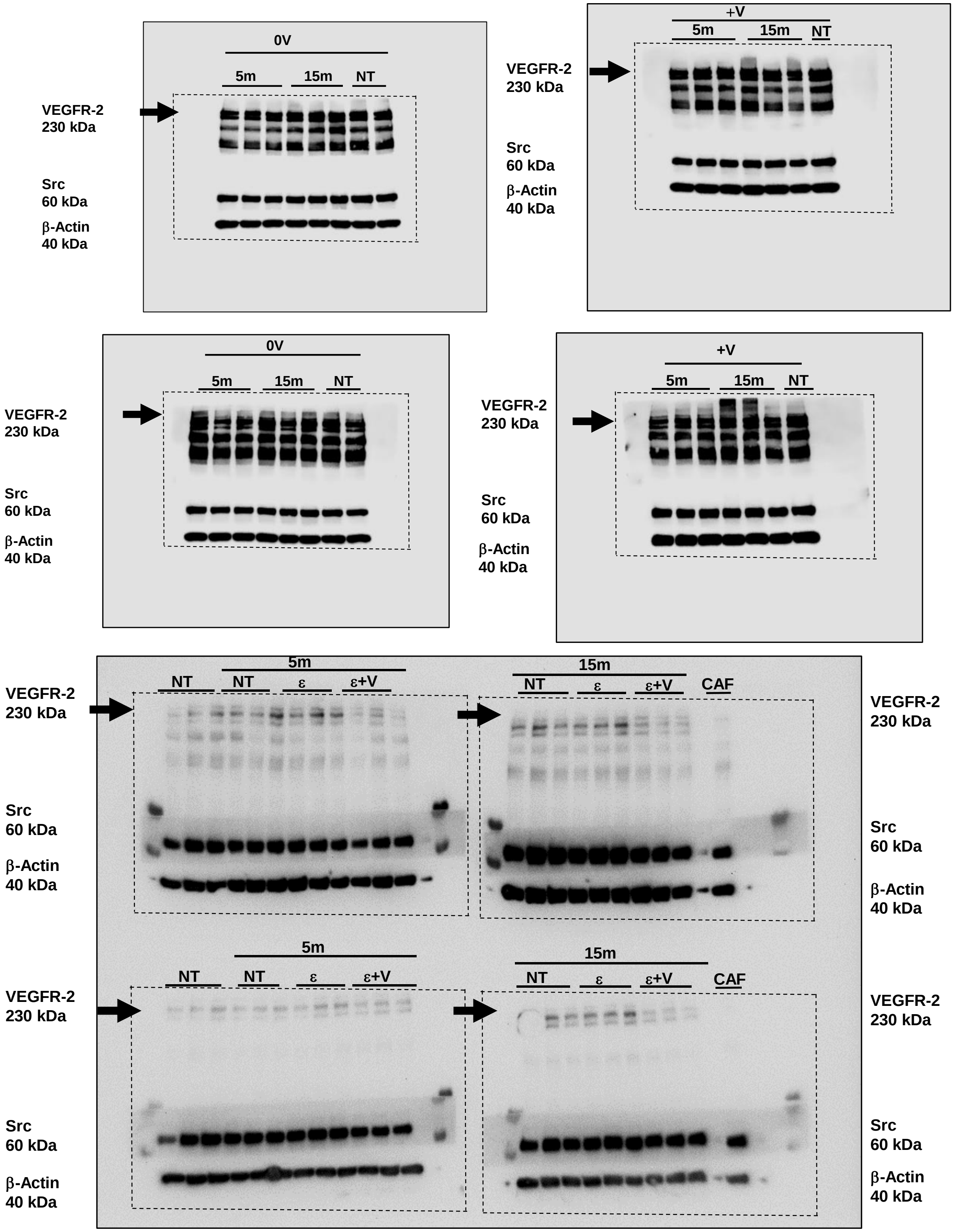
0V
5m
15m
NT
VEGFR-2
230 kDa
Src
60 kDa
β-Actin
40 kDa
+V
5m
15m
NT
VEGFR-2
230 kDa
Src
60 kDa
β-Actin
40 kDa
0V
5m
15m
NT
VEGFR-2
230 kDa
Src
60 kDa
β-Actin
40 kDa
+V
5m
15m
NT
VEGFR-2
230 kDa
Src
60 kDa
β-Actin
40 kDa
5m
NT
NT
ε
ε+V
15m
NT
ε
ε+V
CAF
VEGFR-2
230 kDa
Src
60 kDa
β-Actin
40 kDa
VEGFR-2
230 kDa
Src
60 kDa
β-Actin
40 kDa
5m
NT
NT
ε
ε+V
15m
NT
ε
ε+V
CAF
VEGFR-2
230 kDa
Src
60 kDa
β-Actin
40 kDa
VEGFR-2
230 kDa
Src
60 kDa
β-Actin
40 kDa

2b

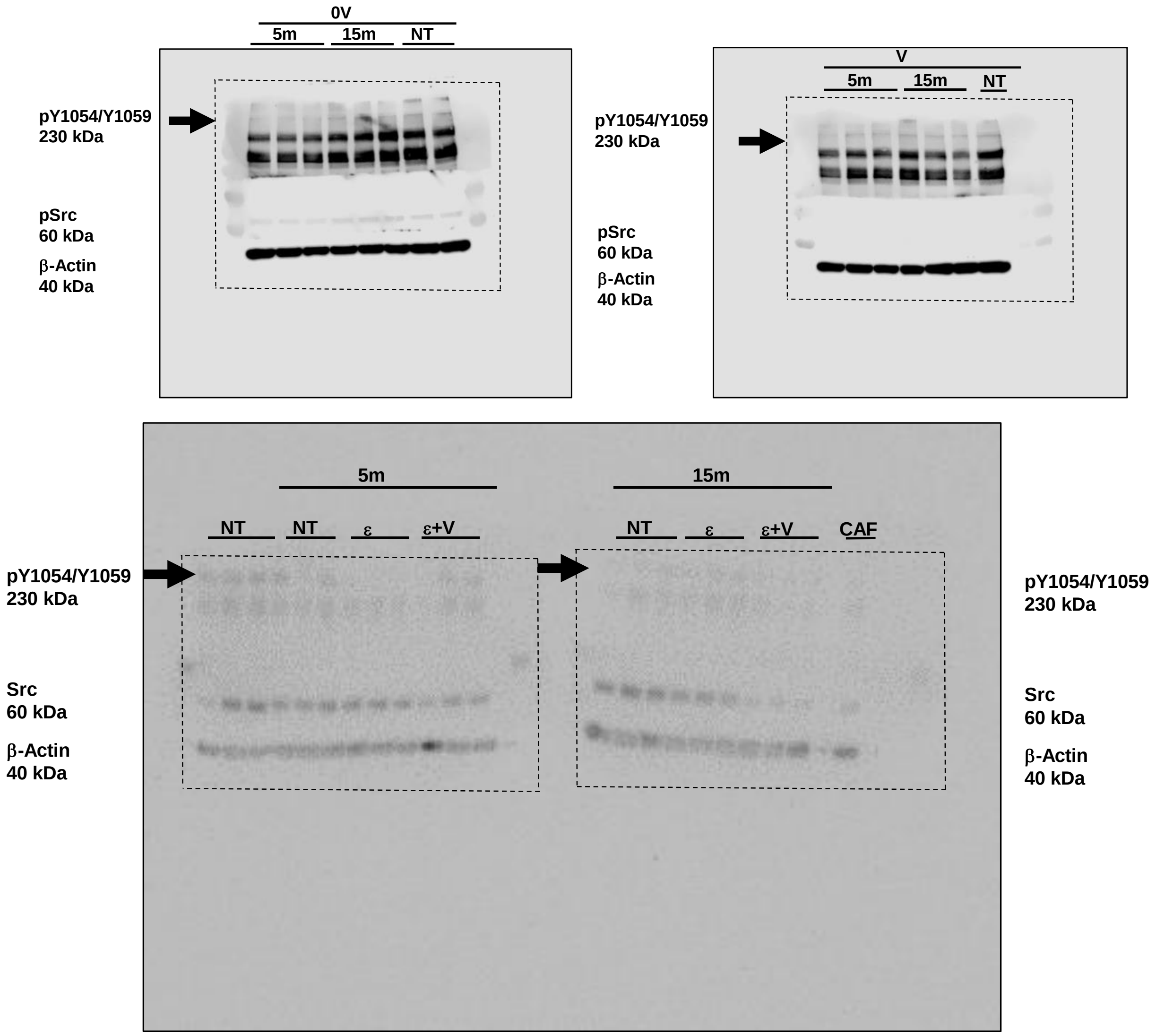
0V
5m
15m
NT
pY1054/Y1059
230 kDa
pSrc
60 kDa
β-Actin
40 kDa
V
5m
15m
NT
pY1054/Y1059
230 kDa
pSrc
60 kDa
β-Actin
40 kDa
5m
15m
NT
NT
ε
ε+V
NT
ε
ε+V
CAF
pY1054/Y1059
230 kDa
Src
60 kDa
β-Actin
40 kDa
pY1054/Y1059
230 kDa
Src
60 kDa
β-Actin
40 kDa

2c

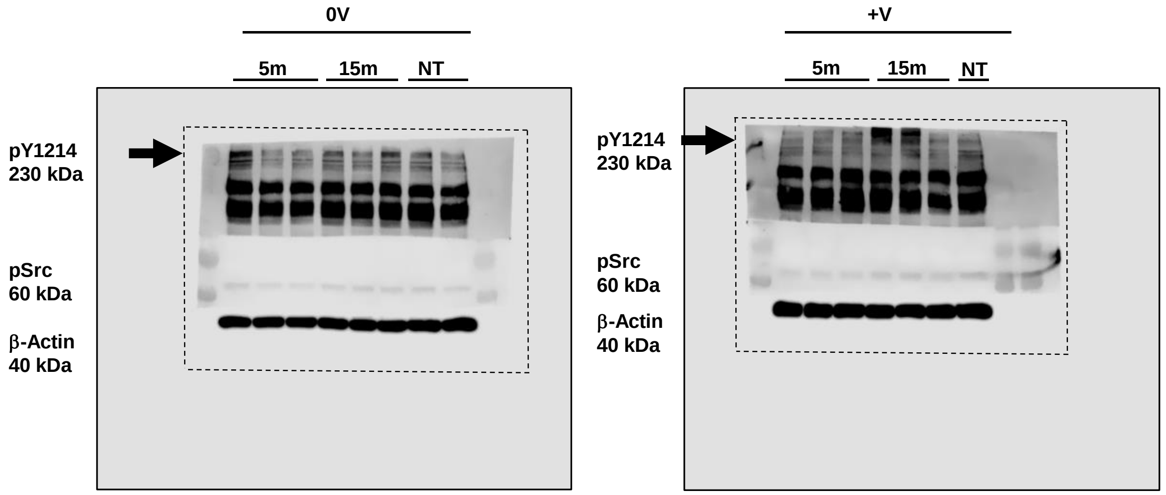
0V
5m
15m
NT
pY1214
230 kDa
pSrc
60 kDa
β-Actin
40 kDa
+V
5m
15m
NT
pY1214
230 kDa
pSrc
60 kDa
β-Actin
40 kDa

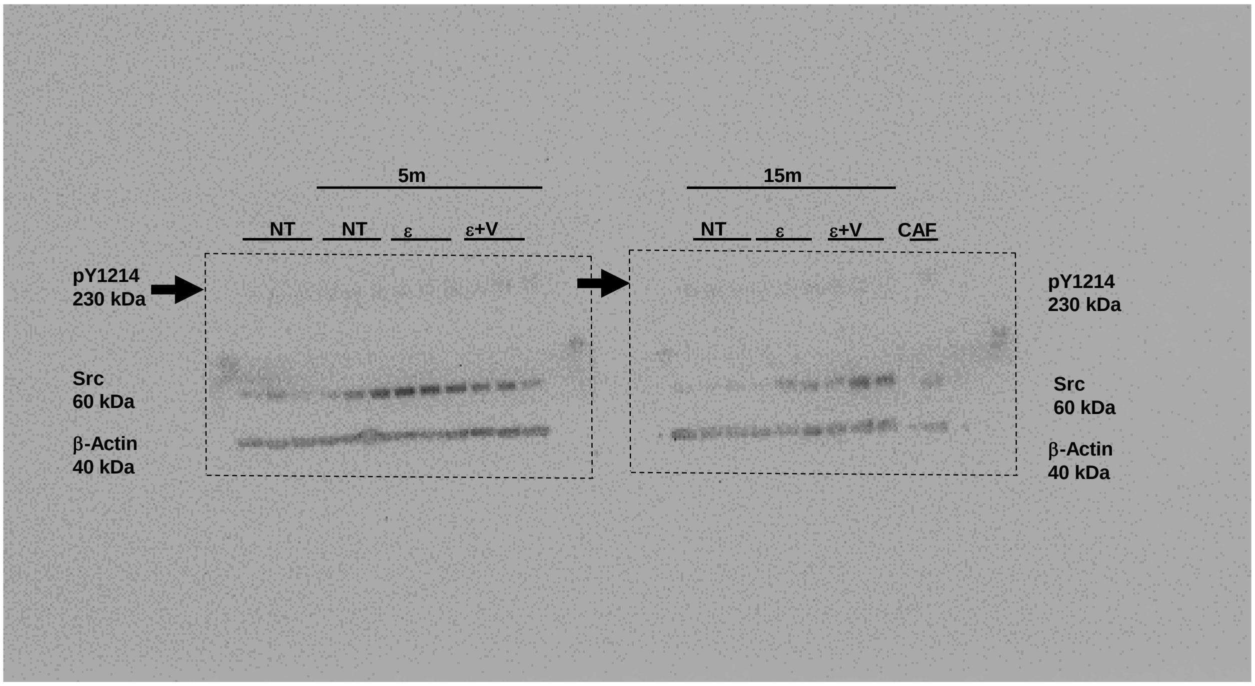
5m
15m
NT
NT
ε
ε+V
NT
ε
ε+V
CAF
pY1214
230 kDa
Src
60 kDa
β-Actin
40 kDa
pY1214
230 kDa
Src
60 kDa
β-Actin
40 kDa

2d

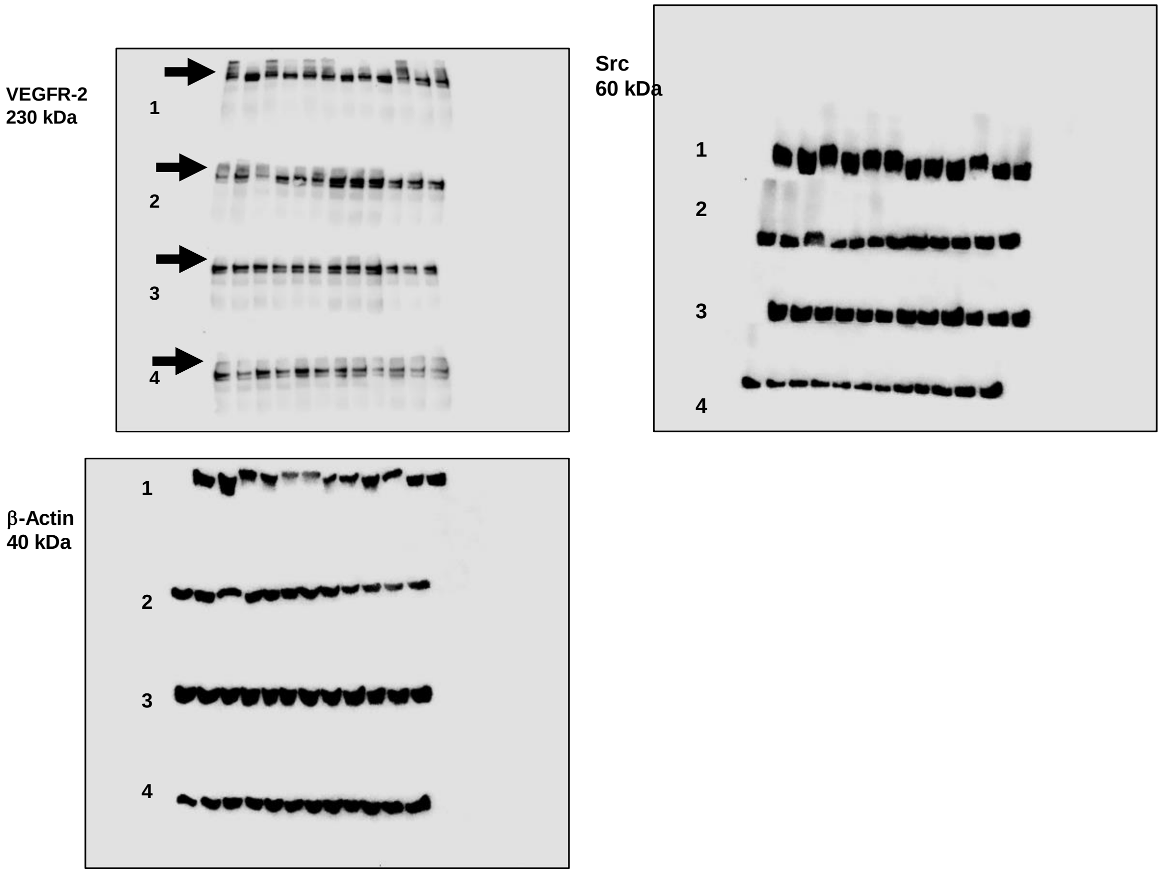
VEGFR-2
230 kDa
1
2
3
4
Src
60 kDa
1
2
3
4
β-Actin
40 kDa
1
2
3
4

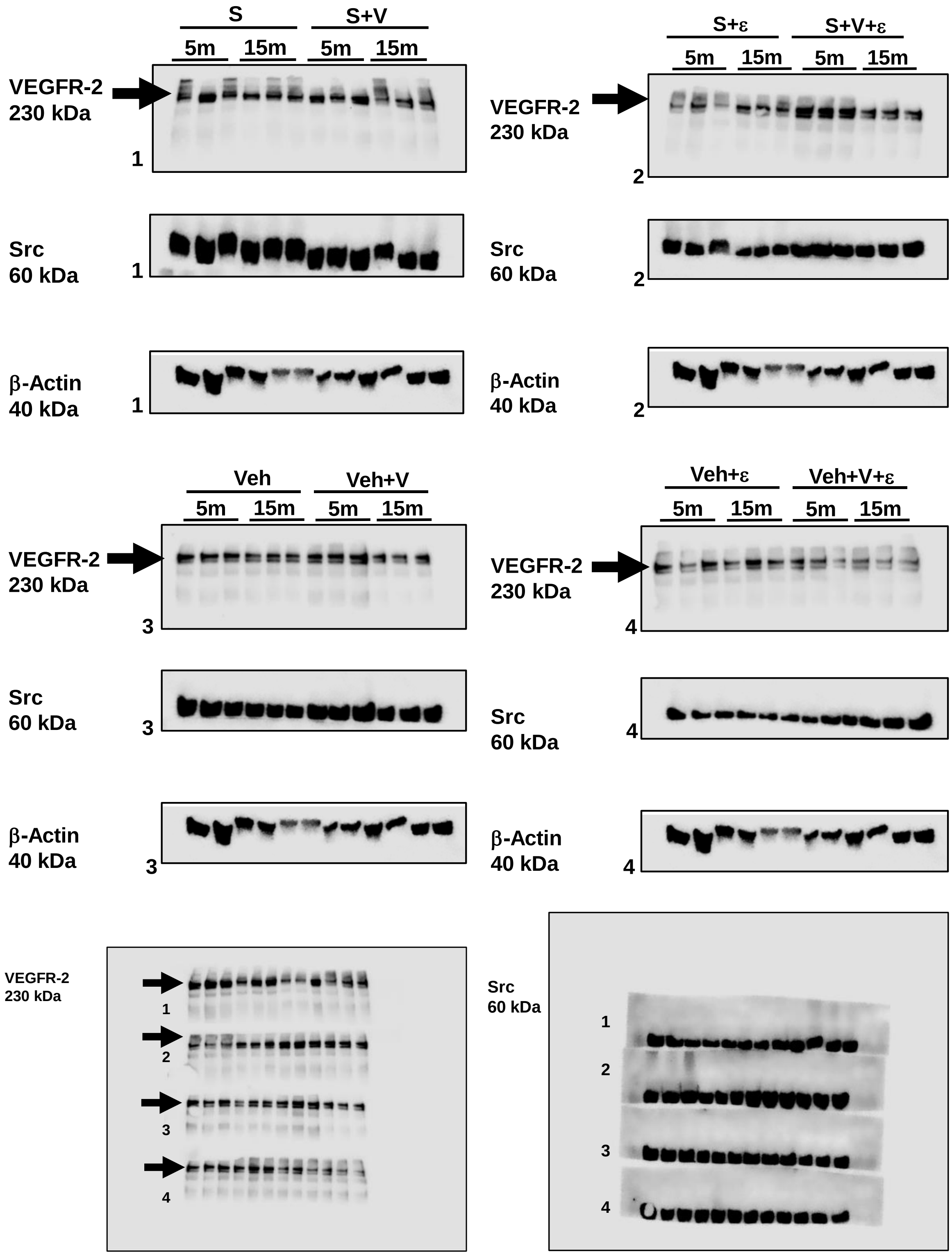
S
S+V
5m
15m
5m
15m
VEGFR-2
230 kDa
1
Src
60 kDa
1
β-Actin
40 kDa
1
S+ε
S+V+ε
5m
15m
5m
15m
VEGFR-2
230 kDa
2
Src
60 kDa
2
β-Actin
40 kDa
2
Veh
Veh+V
5m
15m
5m
15m
VEGFR-2
230 kDa
3
Src
60 kDa
3
β-Actin
40 kDa
3
Veh+ε
Veh+V+ε
5m
15m
5m
15m
VEGFR-2
230 kDa
4
Src
60 kDa
4
β-Actin
40 kDa
4
VEGFR-2
230 kDa
1
2
3
4
Src
60 kDa
1
2
3
4

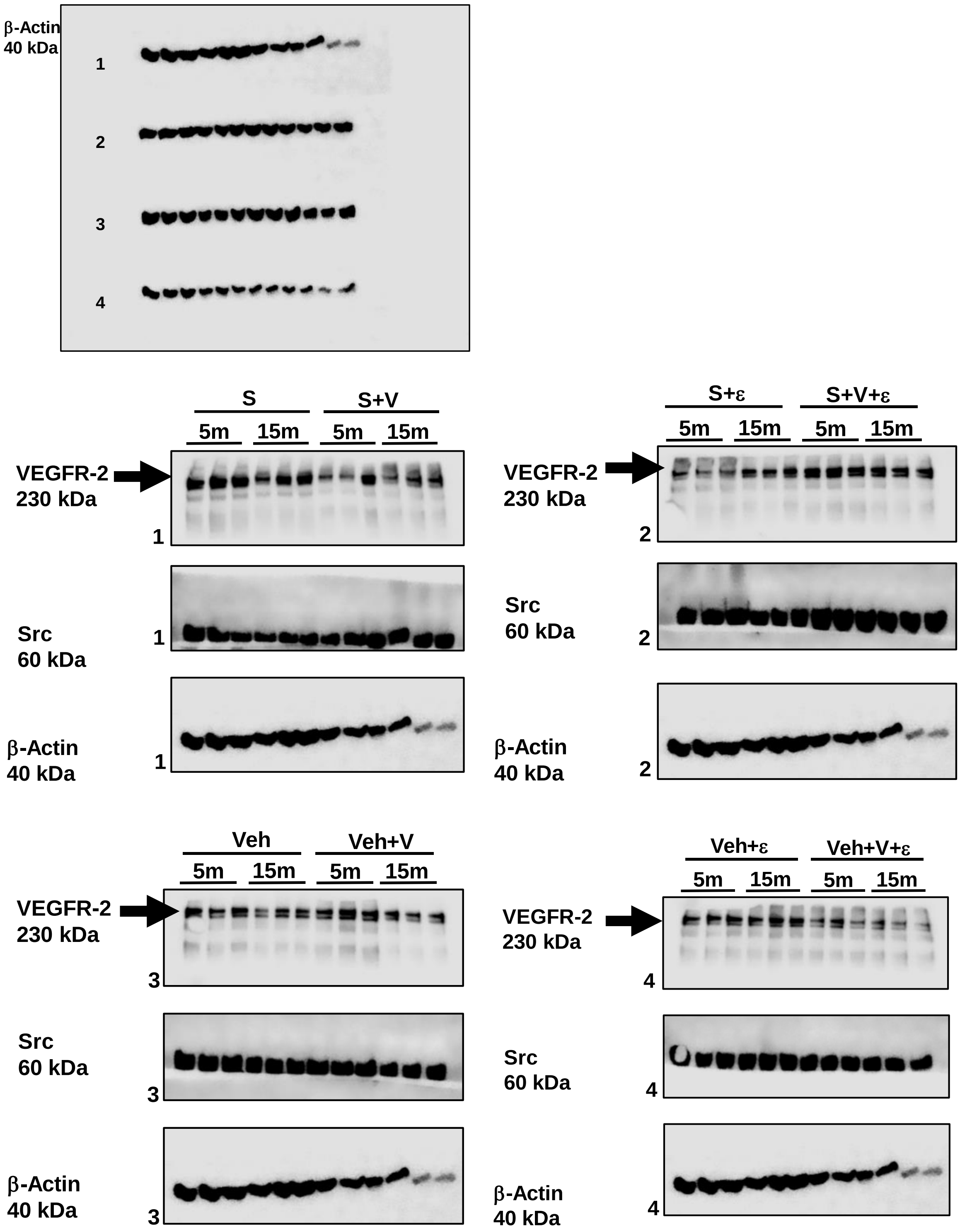
β-Actin
40 kDa
1
2
3
4
S
S+V
5m
15m
5m
15m
VEGFR-2
230 kDa
1
Src
60 kDa
1
β-Actin
40 kDa
1
S+ε
S+V+ε
5m
15m
5m
15m
VEGFR-2
230 kDa
2
Src
60 kDa
2
β-Actin
40 kDa
2
Veh
Veh+V
5m
15m
5m
15m
VEGFR-2
230 kDa
3
Src
60 kDa
3
β-Actin
40 kDa
3
Veh+ε
Veh+V+ε
5m
15m
5m
15m
VEGFR-2
230 kDa
4
Src
60 kDa
4
β-Actin
40 kDa
4

2e

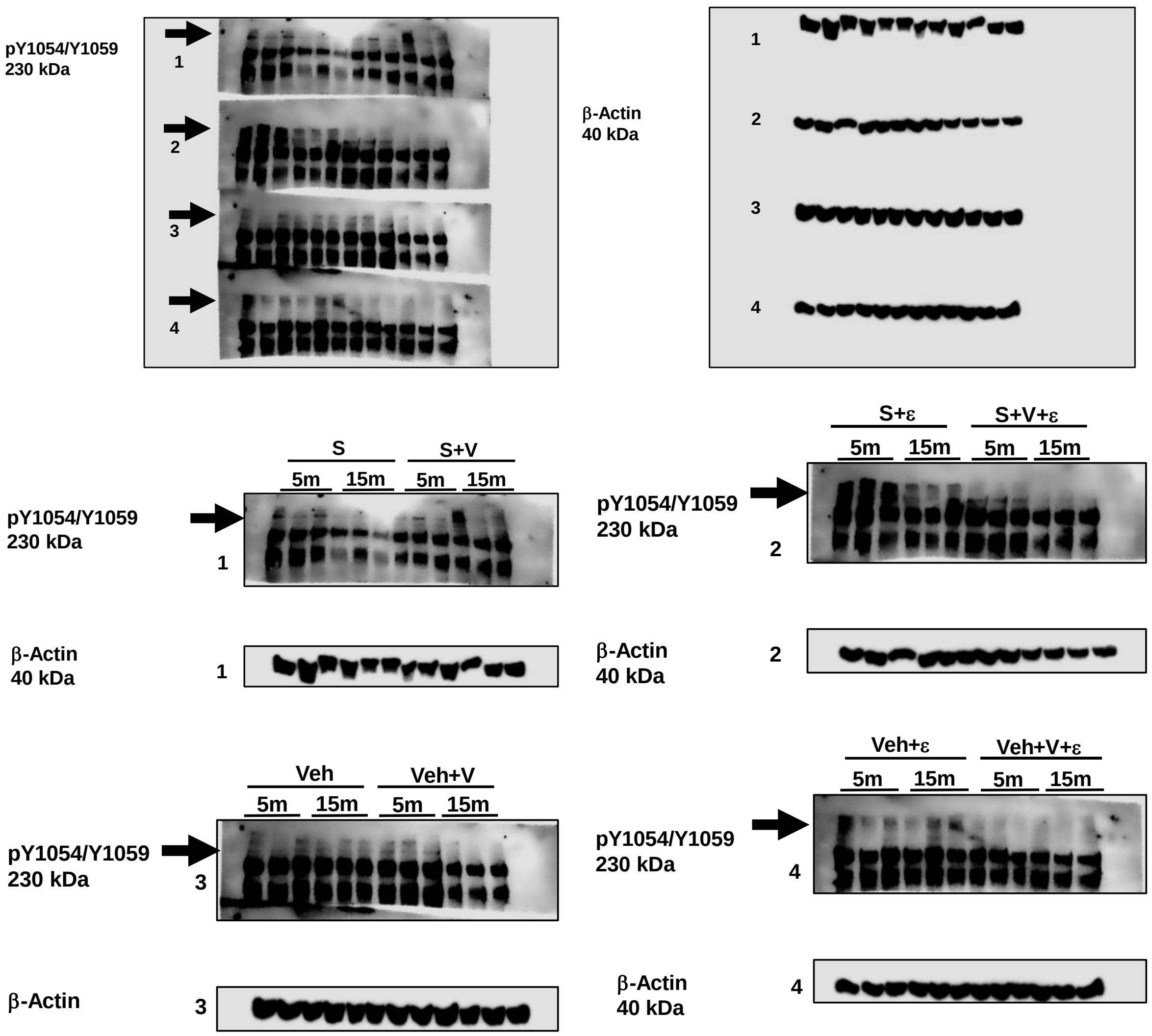
pY1054/Y1059
230 kDa
1
2
3
4
β-Actin
40 kDa
1
2
3
4
S
S+V
5m
15m
5m
15m
pY1054/Y1059
230 kDa
1
β-Actin
40 kDa
1
S+ε
S+V+ε
5m
15m
5m
15m
pY1054/Y1059
230 kDa
2
β-Actin
40 kDa
2
Veh
Veh+V
5m
15m
5m
15m
pY1054/Y1059
230 kDa
3
β-Actin
3
Veh+ε
Veh+V+ε
5m
15m
5m
15m
pY1054/Y1059
230 kDa
4
β-Actin
40 kDa
4

2f

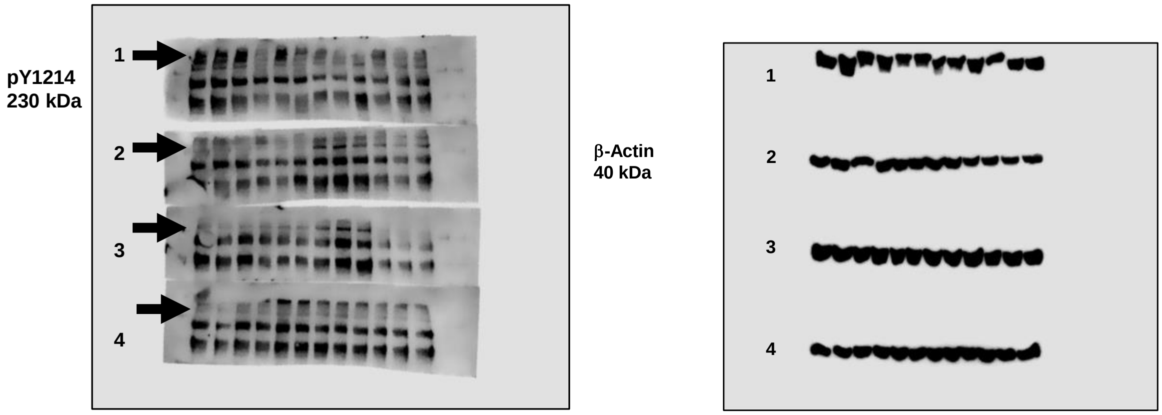
pY1214
230 kDa
1
2
3
4
β-Actin
40 kDa
1
2
3
4

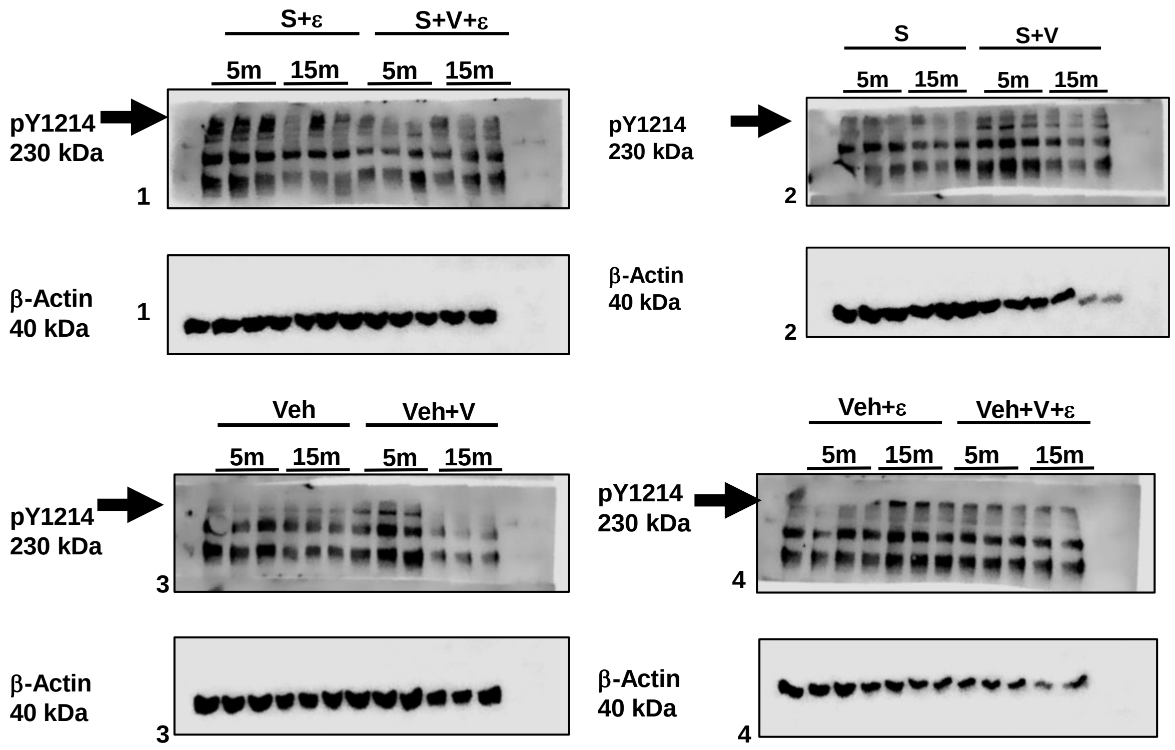
S+ε
S+V+ε
5m
15m
5m
15m
pY1214
230 kDa
1
β-Actin
40 kDa
1
S
S+V
5m
15m
5m
15m
pY1214
230 kDa
2
β-Actin
40 kDa
2
Veh
Veh+V
5m
15m
5m
15m
pY1214
230 kDa
3
β-Actin
40 kDa
3
Veh+ε
Veh+V+ε
5m
15m
5m
15m
pY1214
230 kDa
4
β-Actin
40 kDa
4

2g

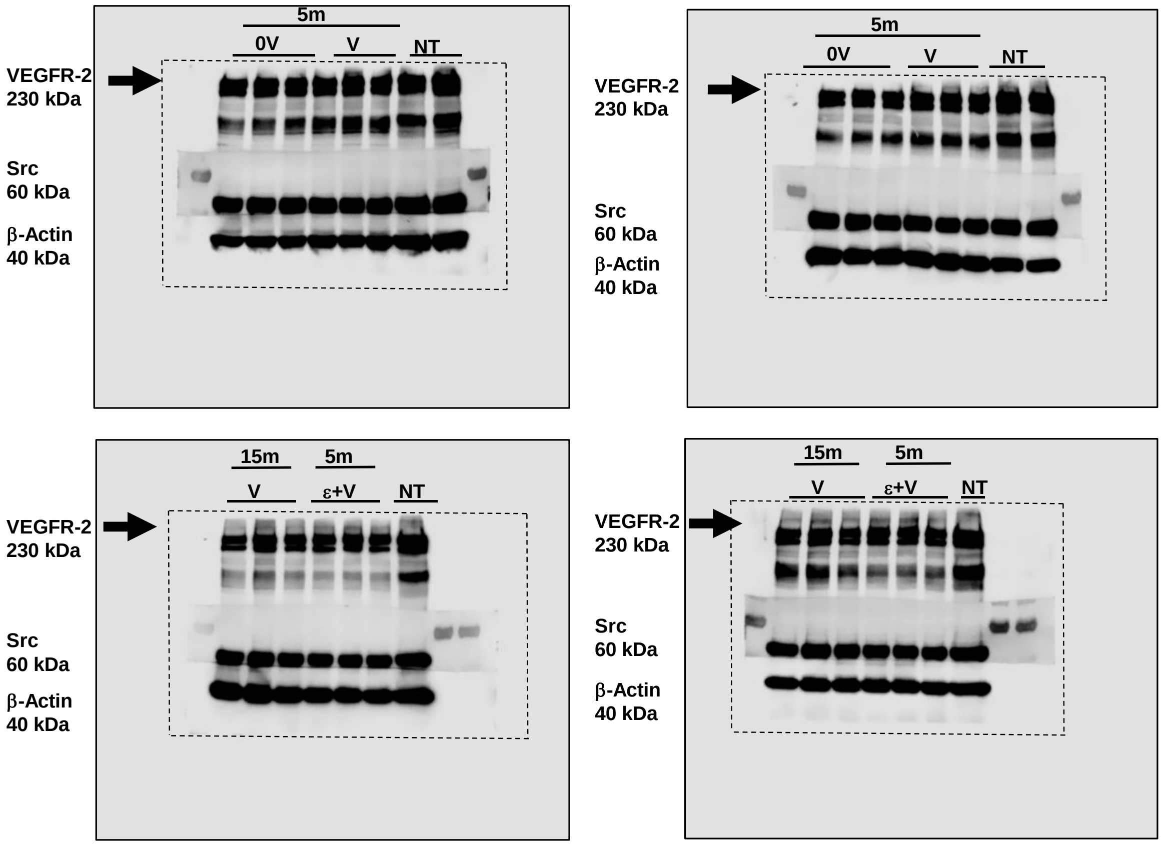
5m
0V
V
NT
VEGFR-2
230 kDa
Src
60 kDa
β-Actin
40 kDa
5m
0V
V
NT
VEGFR-2
230 kDa
Src
60 kDa
β-Actin
40 kDa
15m
5m
V
ε+V
NT
VEGFR-2
230 kDa
Src
60 kDa
β-Actin
40 kDa
15m
5m
V
ε+V
NT
VEGFR-2
230 kDa
Src
60 kDa
β-Actin
40 kDa

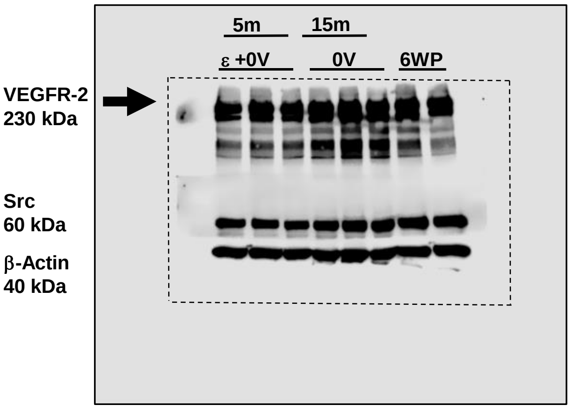
5m
15m
ε +0V
0V
6WP
VEGFR-2
230 kDa
Src
60 kDa
β-Actin
40 kDa

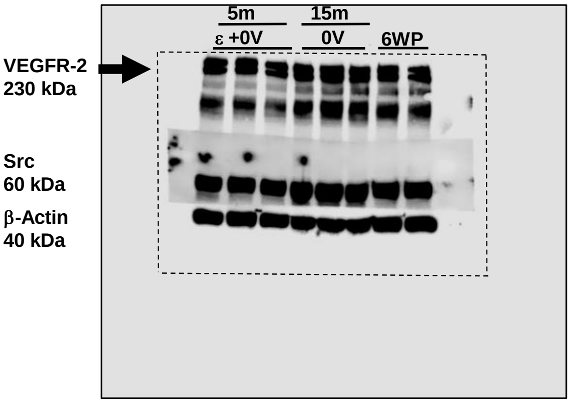
5m
15m
ε +0V
0V
6WP
VEGFR-2
230 kDa
Src
60 kDa
β-Actin
40 kDa

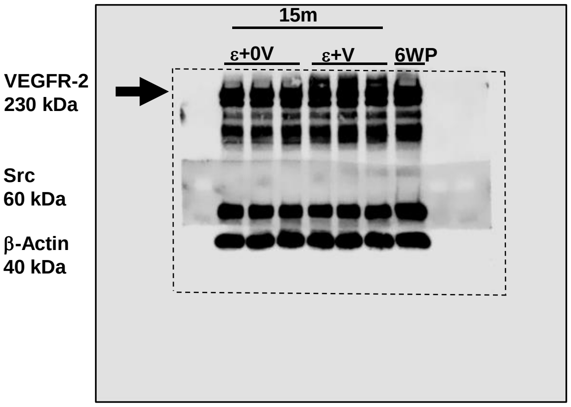
15m
ε+0V
ε+V
6WP
VEGFR-2
230 kDa
Src
60 kDa
β-Actin
40 kDa

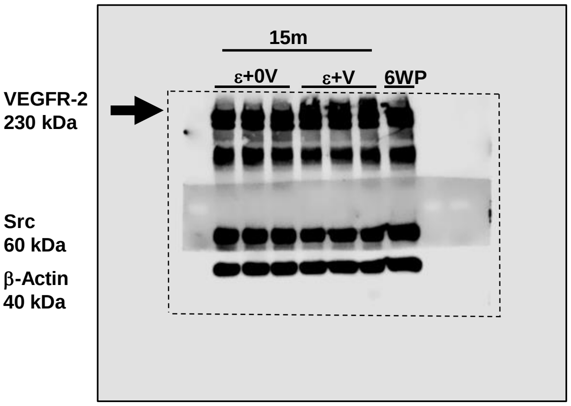
15m
ε+0V
ε+V
6WP
VEGFR-2
230 kDa
Src
60 kDa
β-Actin
40 kDa

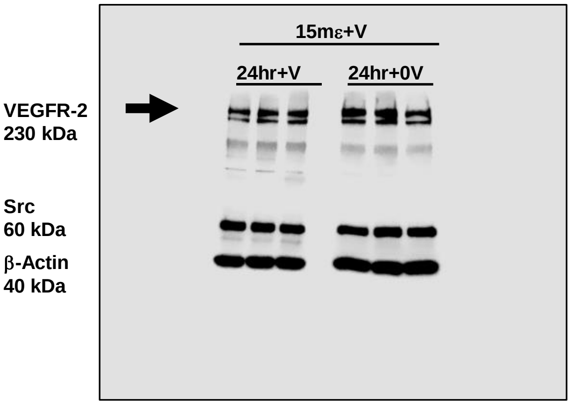
15mε+V
24hr+V
24hr+0V
VEGFR-2
230 kDa
Src
60 kDa
β-Actin
40 kDa

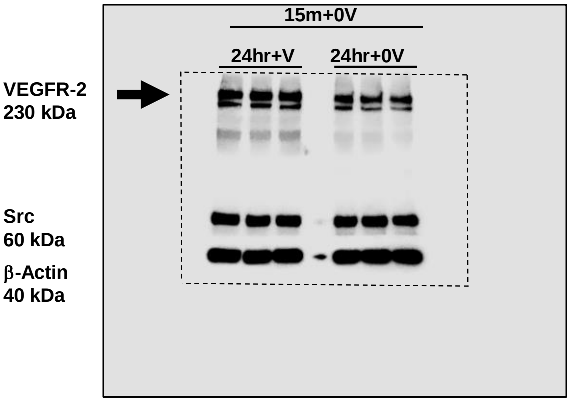
15m+0V
24hr+V
24hr+0V
VEGFR-2
230 kDa
Src
60 kDa
β-Actin
40 kDa

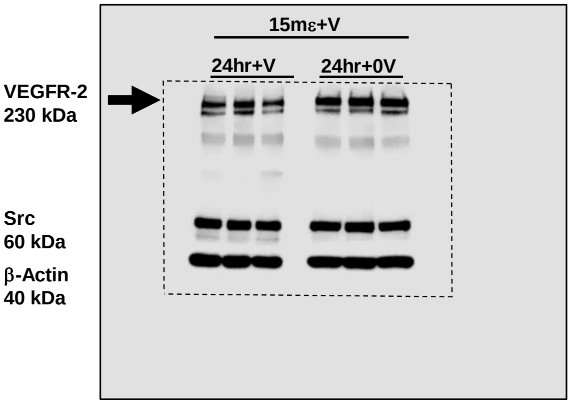
15mε+V
24hr+V
24hr+0V
VEGFR-2
230 kDa
Src
60 kDa
β-Actin
40 kDa

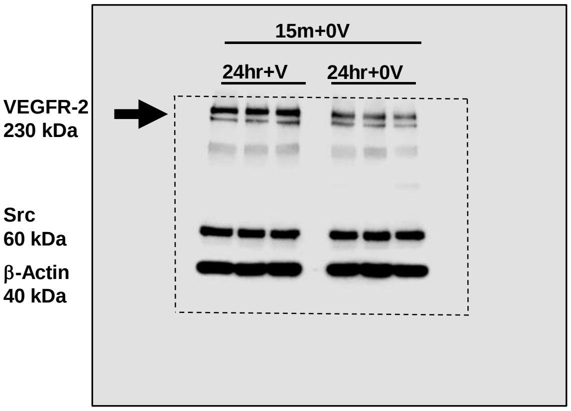
15m+0V
24hr+V
24hr+0V
VEGFR-2
230 kDa
Src
60 kDa
β-Actin
40 kDa

2h

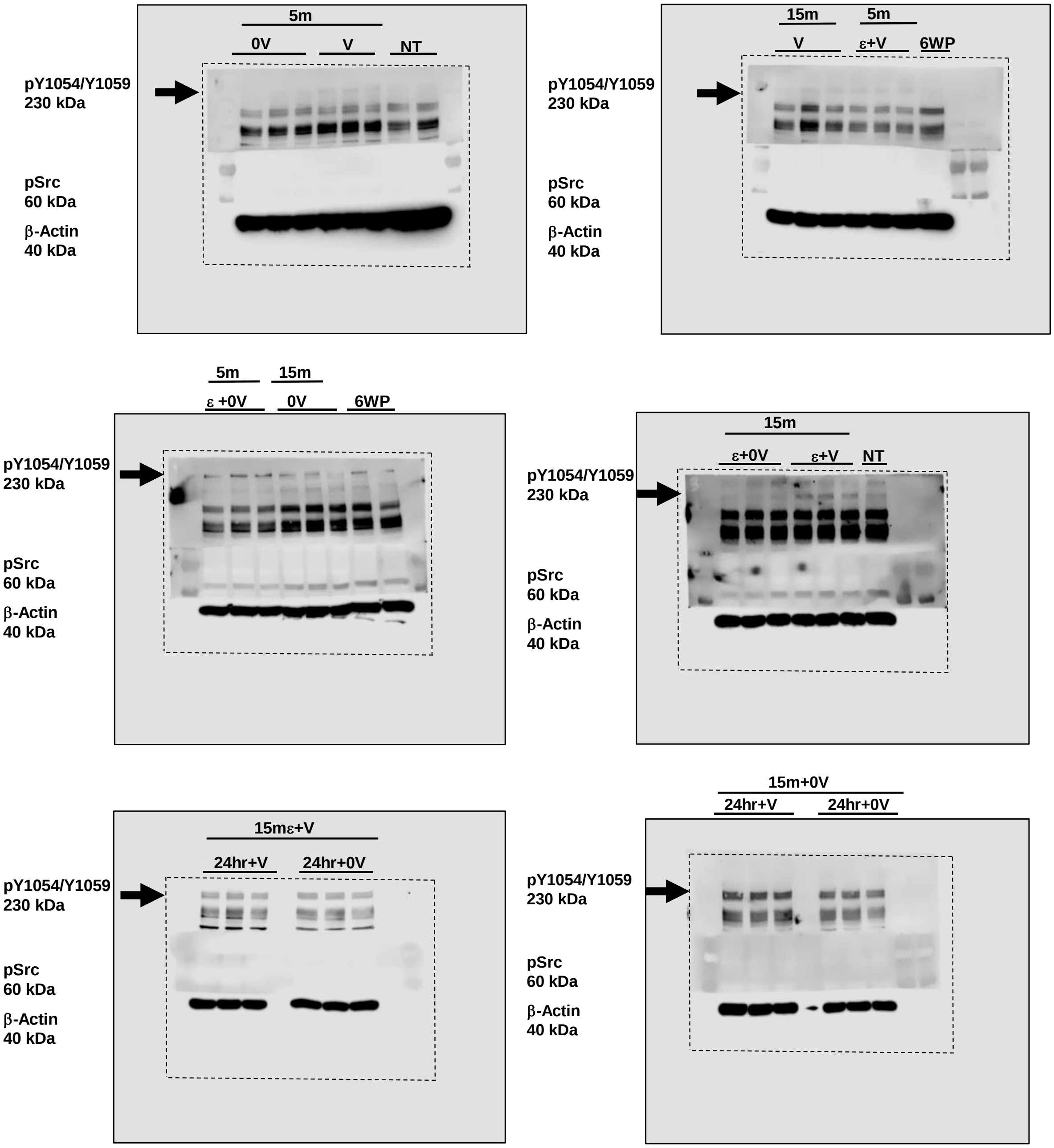
5m
0V
V
NT
pY1054/Y1059
230 kDa
pSrc
60 kDa
β-Actin
40 kDa
15m
5m
V
ε+V
6WP
pY1054/Y1059
230 kDa
pSrc
60 kDa
β-Actin
40 kDa
5m
15m
ε +0V
0V
6WP
pY1054/Y1059
230 kDa
pSrc
60 kDa
β-Actin
40 kDa
15m
ε+0V
ε+V
NT
pY1054/Y1059
230 kDa
pSrc
60 kDa
β-Actin
40 kDa
15mε+V
24hr+V
24hr+0V
pY1054/Y1059
230 kDa
pSrc
60 kDa
β-Actin
40 kDa
15m+0V
24hr+V
24hr+0V
pY1054/Y1059
230 kDa
pSrc
60 kDa
β-Actin
40 kDa

2i

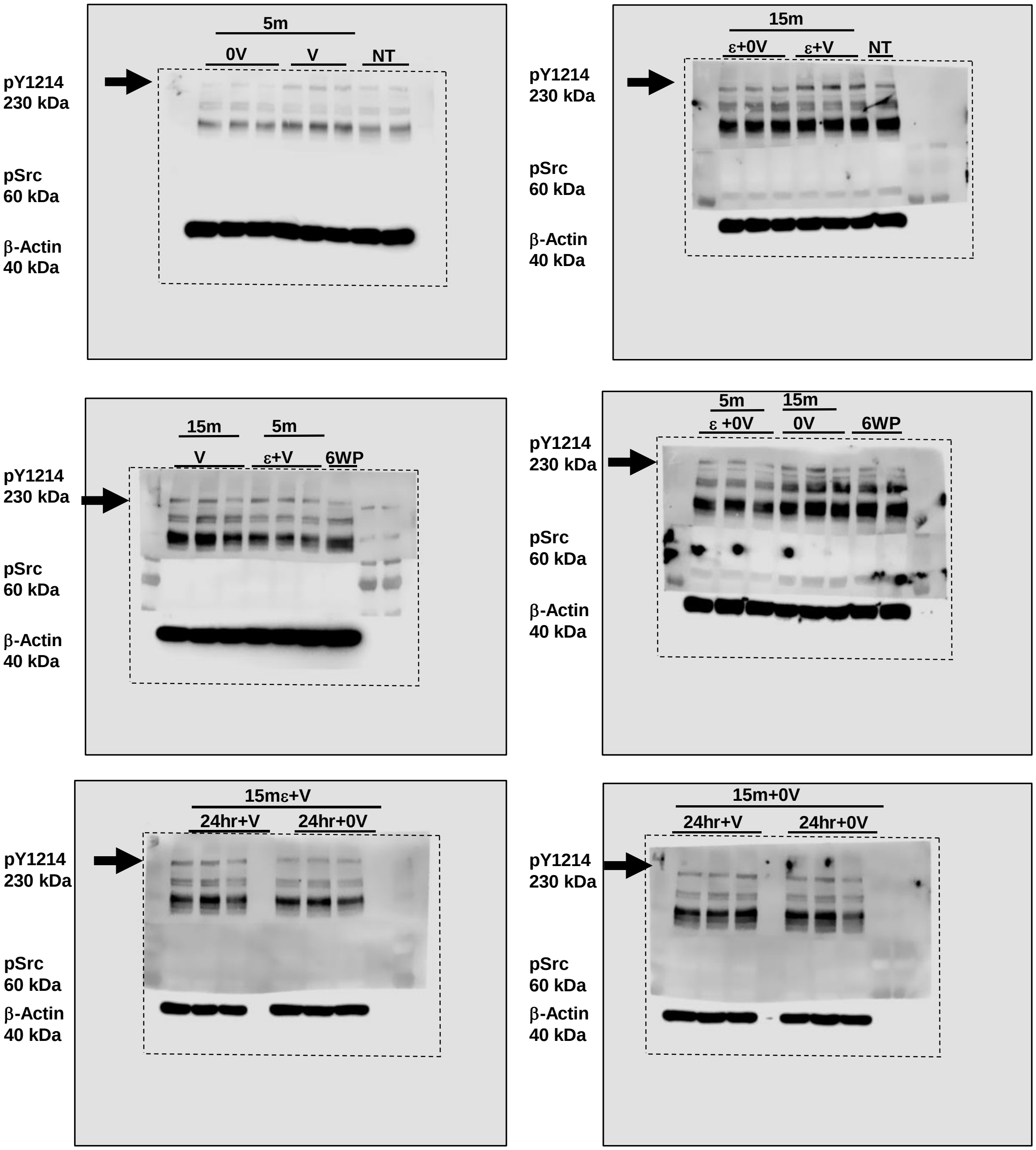
5m
0V
V
NT
pY1214
230 kDa
pSrc
60 kDa
β-Actin
40 kDa
15m
ε+0V
ε+V
NT
pY1214
230 kDa
pSrc
60 kDa
β-Actin
40 kDa
15m
5m
V
ε+V
6WP
pY1214
230 kDa
pSrc
60 kDa
β-Actin
40 kDa
5m
15m
ε +0V
0V
6WP
pY1214
230 kDa
pSrc
60 kDa
β-Actin
40 kDa
15mε+V
24hr+V
24hr+0V
pY1214
230 kDa
pSrc
60 kDa
β-Actin
40 kDa
15m+0V
24hr+V
24hr+0V
pY1214
230 kDa
pSrc
60 kDa
β-Actin
40 kDa

2j

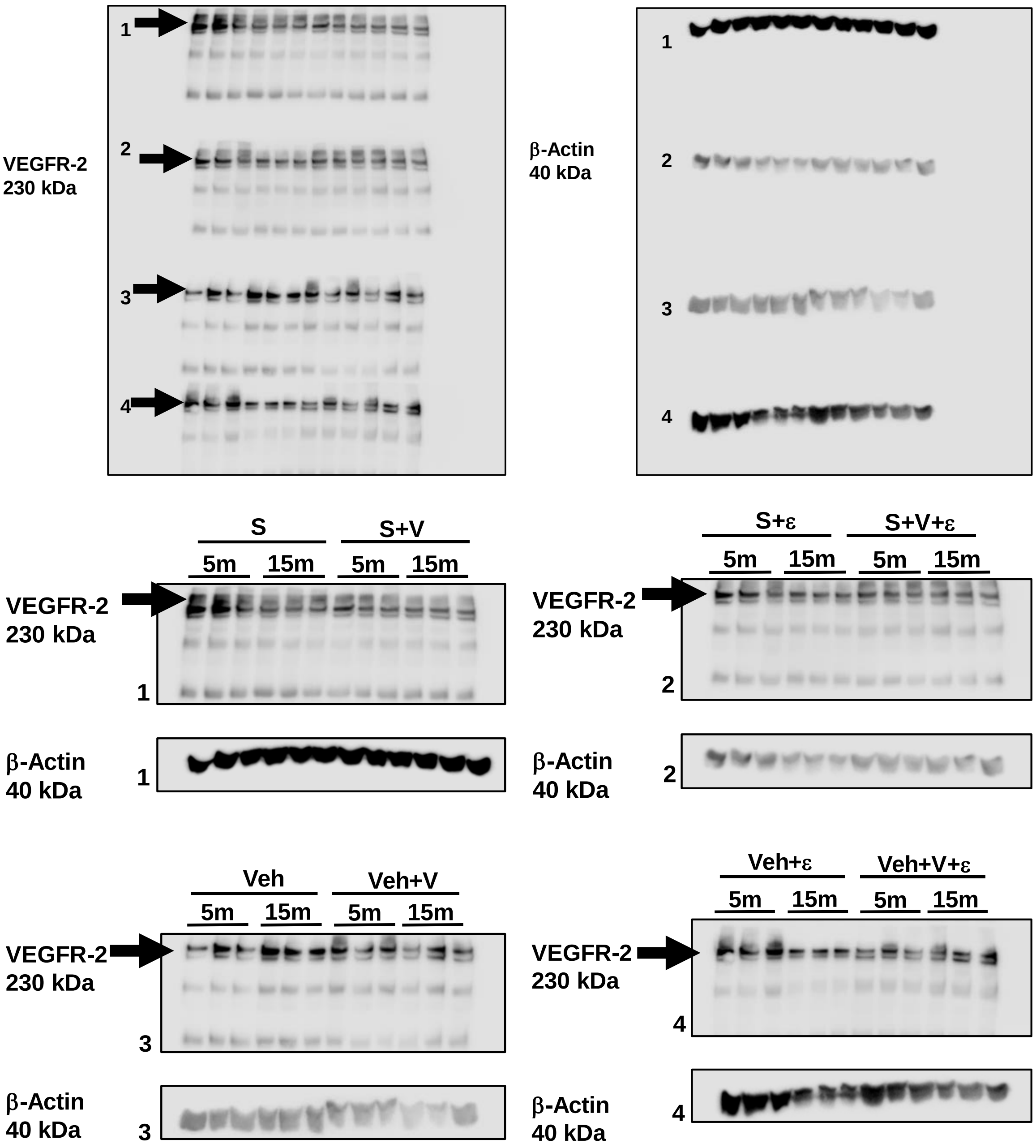
VEGFR-2
230 kDa
β-Actin
40 kDa
1
2
3
4
S
S+V
5m
15m
S+ε
S+V+ε
Veh
Veh+V
Veh+ε
Veh+V+ε

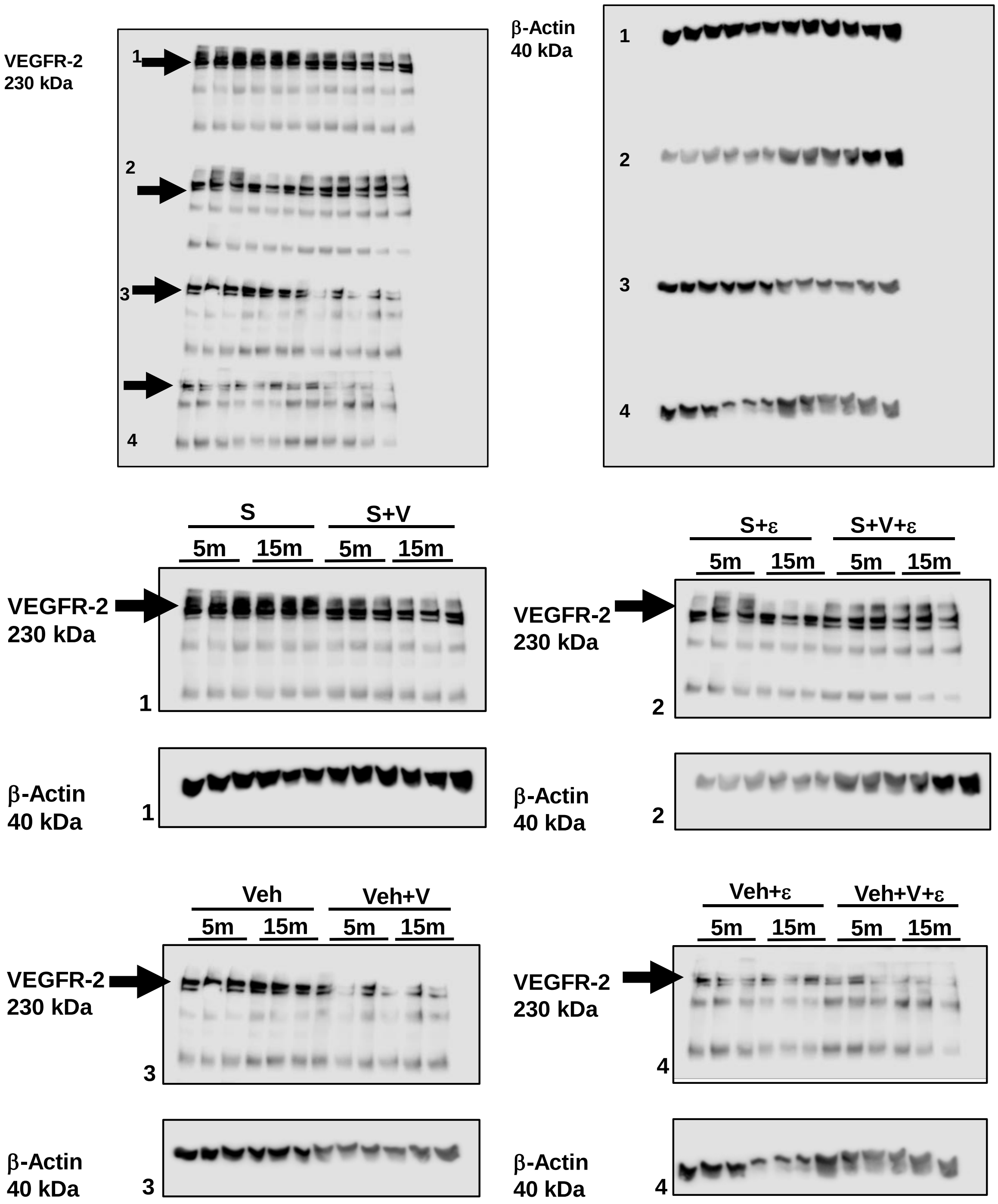
VEGFR-2
230 kDa
β-Actin
40 kDa
1
2
3
4
S
S+V
5m
15m
S+ε
S+V+ε
Veh
Veh+V
Veh+ε
Veh+V+ε

2k

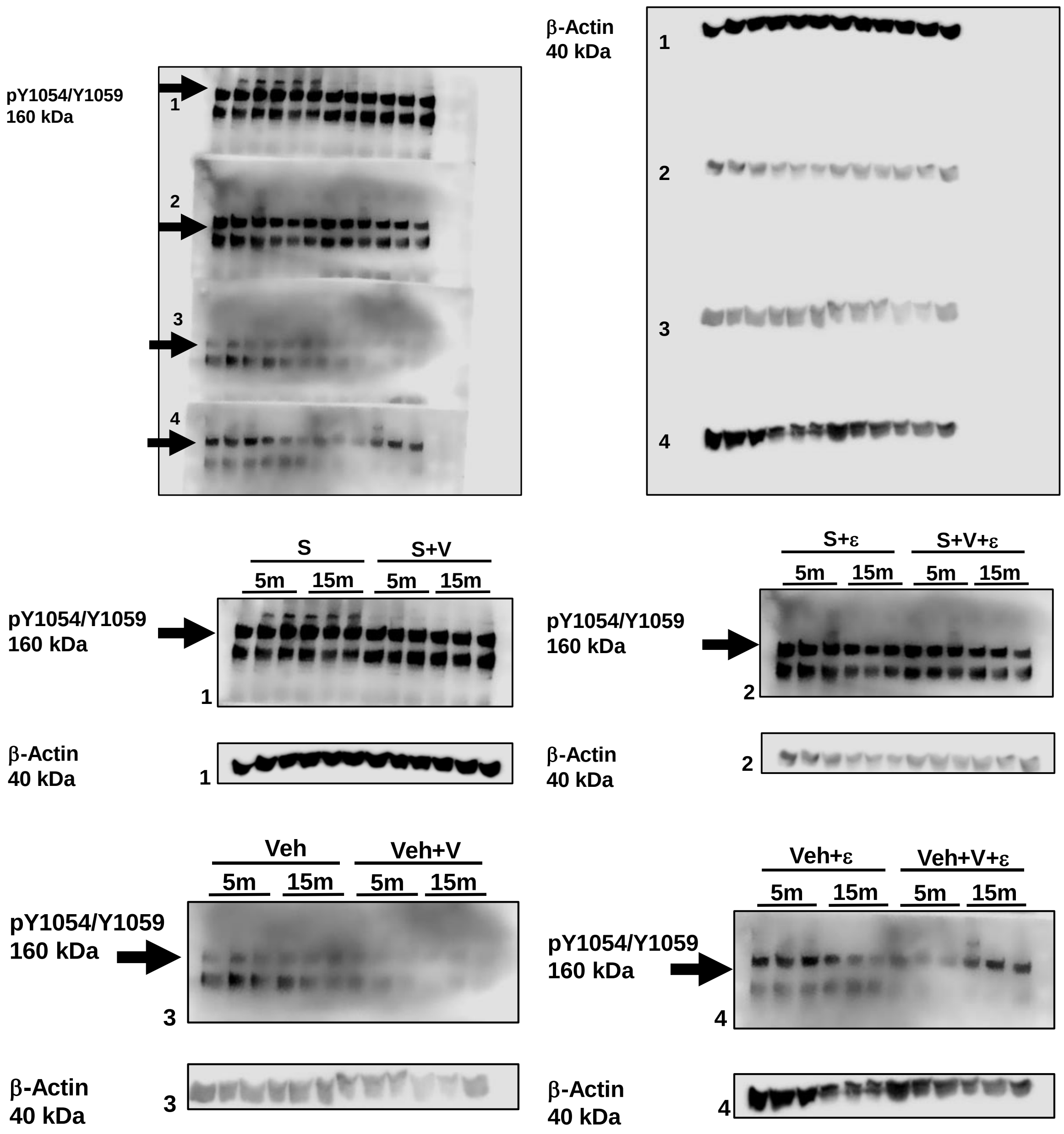
pY1054/Y1059
160 kDa
1
2
3
4
β-Actin
40 kDa
1
2
3
4
S
S+V
5m
15m
5m
15m
pY1054/Y1059
160 kDa
1
β-Actin
40 kDa
1
S+ε
S+V+ε
5m
15m
5m
15m
pY1054/Y1059
160 kDa
2
β-Actin
40 kDa
2
Veh
Veh+V
5m
15m
5m
15m
pY1054/Y1059
160 kDa
3
β-Actin
40 kDa
3
Veh+ε
Veh+V+ε
5m
15m
5m
15m
pY1054/Y1059
160 kDa
4
β-Actin
40 kDa
4

2l

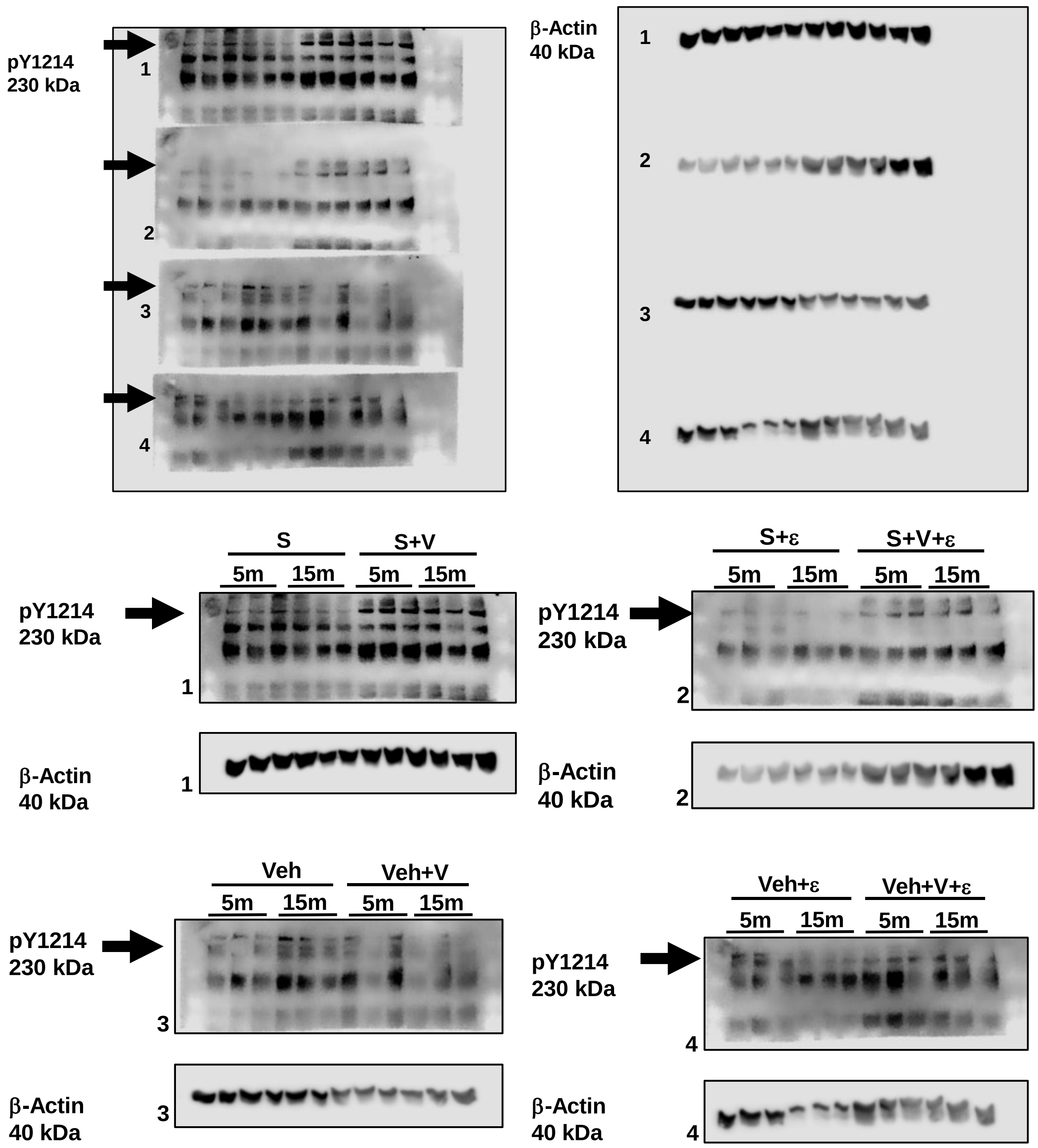

Supplemental Fig. 2 – Labelled Western Blots for Data in Fig. 2. This figure matches Fig. 2 in the main text. **(a)** HMECs were subjected to control (NT) conditions without strain and VEGF, VEGF only (25ng/mL, V), strain only (ε), or strain plus VEGF (ε +V) for 5min or 15min. Western blots were performed to analyze total VEGFR-2, **(b)** pY1054/Y1059, or **(c)** pY1214 levels relative to the 0min, NT control groups. **(d)** HMECs were treated with combinations of strain, VEGF, SU5416, and a vehicle control then stained for total VEGFR-2, **(e)** pY1054/Y1059, and **(f)** pY1214. Some blots were treated with 24hr regular EGM-2 or 0ng/ml VEGF EGM-2 before final treatment to ensure this did not alter results. **(g-l)** HUVECs received the same treatments and stains. All blots were also stained for β-actin as a loading control. Arrows indicated VEGFR-2 bands quantified. Full images are numbered and correspond to labelled pictures.

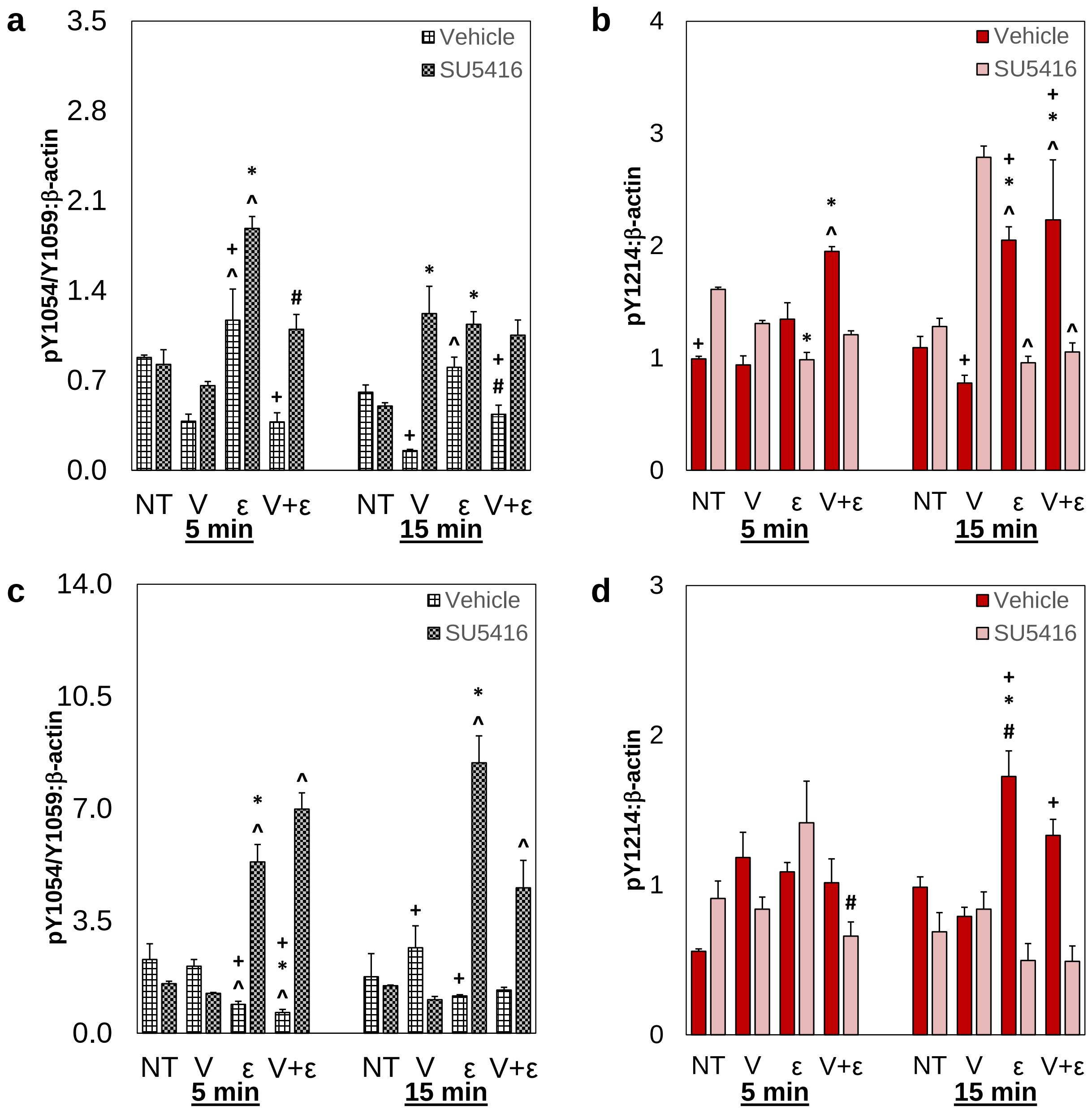

3

Supplemental Fig. 3 – Quantification of pY1054/Y1059 and pY1214 Contrasting Data in Fig. 2e,f,k,l. This data is normalized to β-actin as opposed to total VEGFR-2. **(a)** HMECs were treated with combinations of strain, VEGF (25ng/ml), SU5416 (3μM), and a vehicle control then stained for pY1054/Y1059 and **(b)** pY1214. **(c-d)** Quantification of Western blots for phosphorylated VEGFR-2 in HUVECs with same treatment groups outlined above. Western blot quantifications were normalized to β-actin. pY1054/Y1059 was quantified for the 160kDa cleavage product due to partial absence of the mature product in the blot for part c. For all studies, N =3 with averages + SEM shown. + $p < 0.05$ versus SU5416 with same treatment, * $p<0.05$ compared to NT at same time point, ^ $p < 0.05$ versus V at same time point, # $p < 0.05$ compared to ε at same time point. Groups b and c were compared with Kruskal-Wallis test, followed by post-hoc Dunn's tests. Groups a and d were compared with ANOVA, followed by post-hoc Tukey HSD tests.

4a

pY1054/Y1059
230 kDa

Src
60 kDa

β-Actin
40 kDa

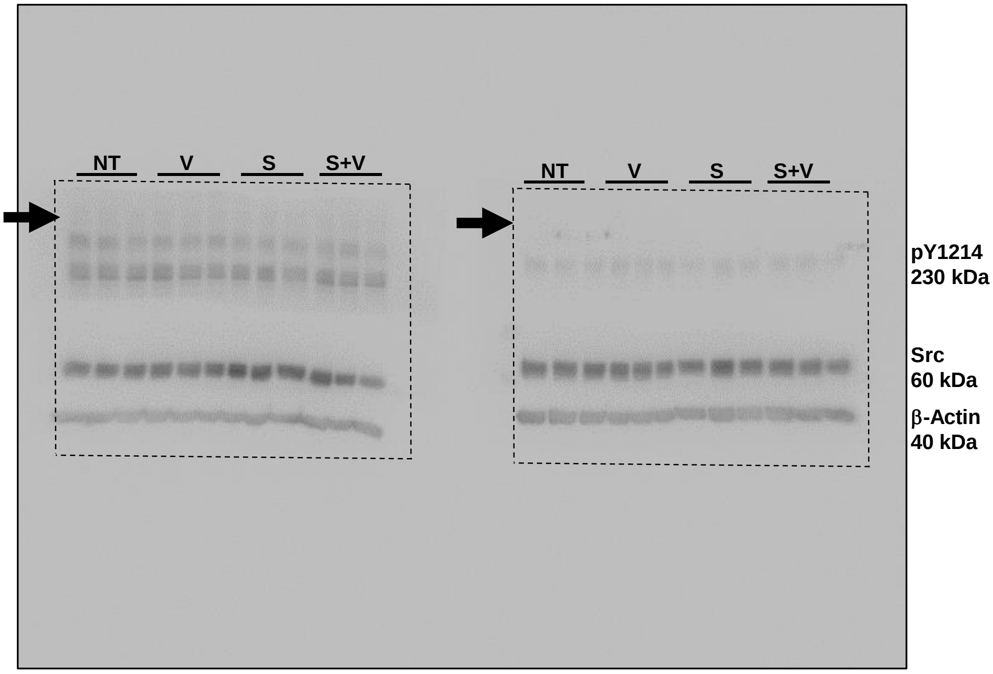

pY1214
230 kDa

Src
60 kDa

β-Actin
40 kDa

4b

pY1054/Y1059
230 kDa

Src
60 kDa

β-Actin
40 kDa

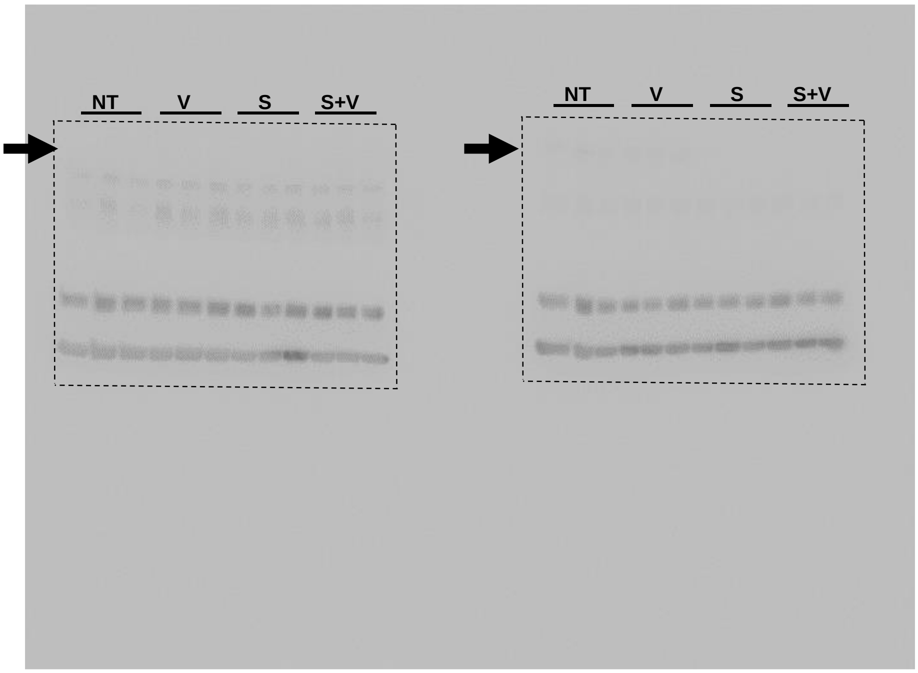

pY1214
230 kDa

Src
60 kDa

β-Actin
40 kDa

4c

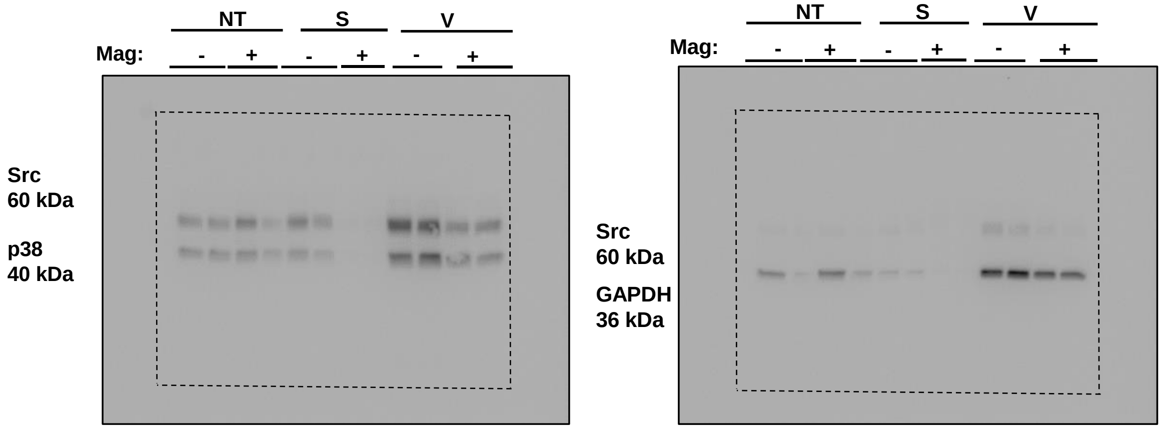
NT
S
V
Mag:
-
+
-
+
-
+
Src
60 kDa
p38
40 kDa
NT
S
V
Mag:
-
+
-
+
-
+
Src
60 kDa
GAPDH
36 kDa

4d

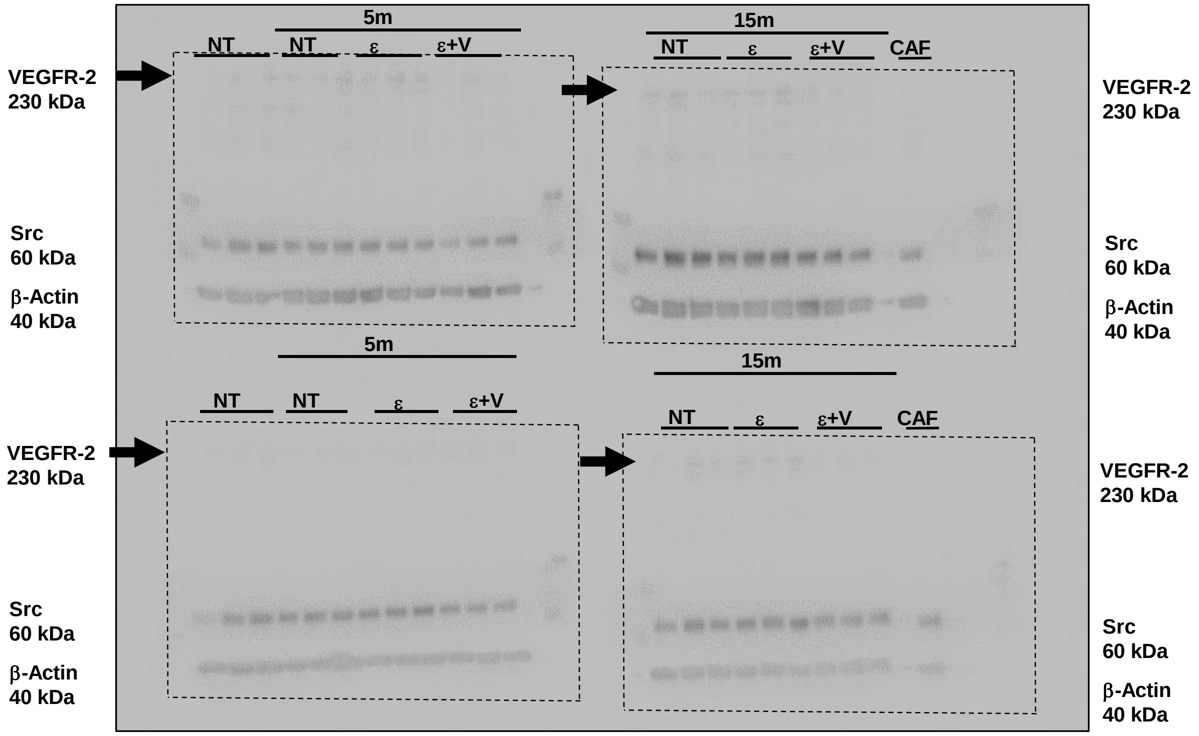
5m
NT
NT
ε
ε+V
VEGFR-2
230 kDa
Src
60 kDa
β-Actin
40 kDa
15m
NT
ε
ε+V
CAF
VEGFR-2
230 kDa
Src
60 kDa
β-Actin
40 kDa
5m
NT
NT
ε
ε+V
VEGFR-2
230 kDa
Src
60 kDa
β-Actin
40 kDa
15m
NT
ε
ε+V
CAF
VEGFR-2
230 kDa
Src
60 kDa
β-Actin
40 kDa

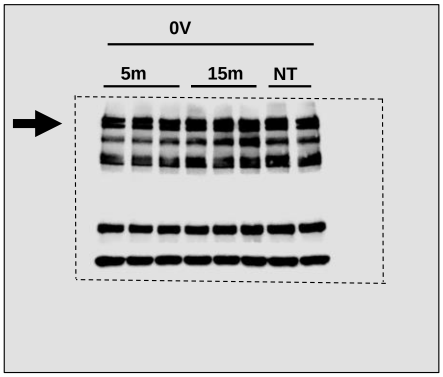
0V
5m
15m
NT
VEGFR-2
230 kDa
Src
60 kDa
β-Actin
40 kDa

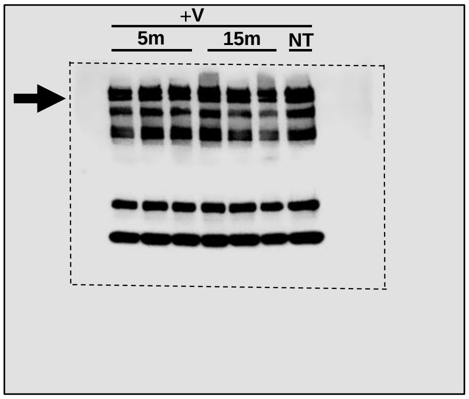
+V
5m
15m
NT
VEGFR-2
230 kDa
Src
60 kDa
β-Actin
40 kDa

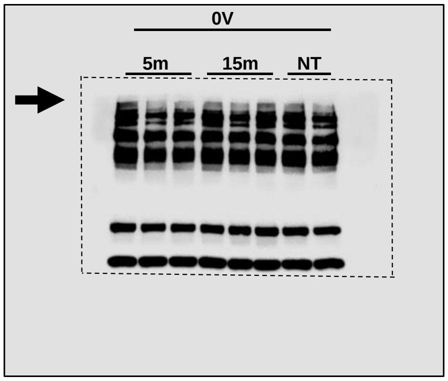
0V
5m
15m
NT
VEGFR-2
230 kDa
Src
60 kDa
β-Actin
40 kDa

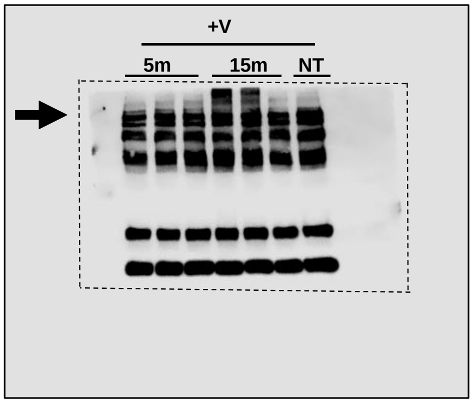
+V
5m
15m
NT
VEGFR-2
230 kDa
Src
60 kDa
β-Actin
40 kDa

4e

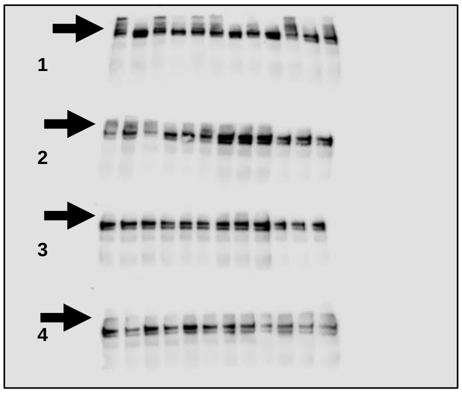
VEGFR-2
230 kDa
1
2
3
4

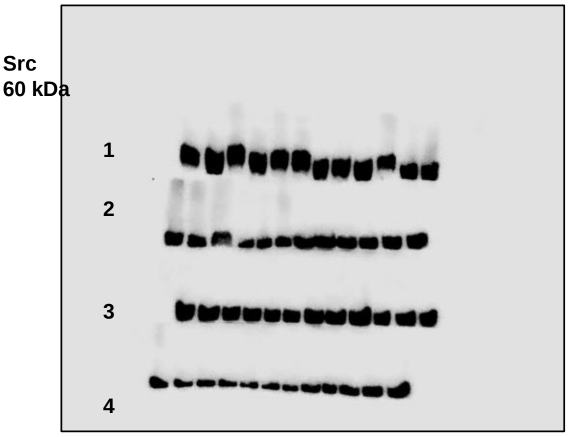
Src
60 kDa
1
2
3
4

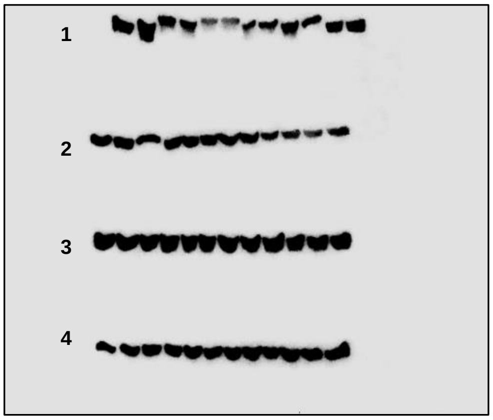
β-Actin
40 kDa
1
2
3
4

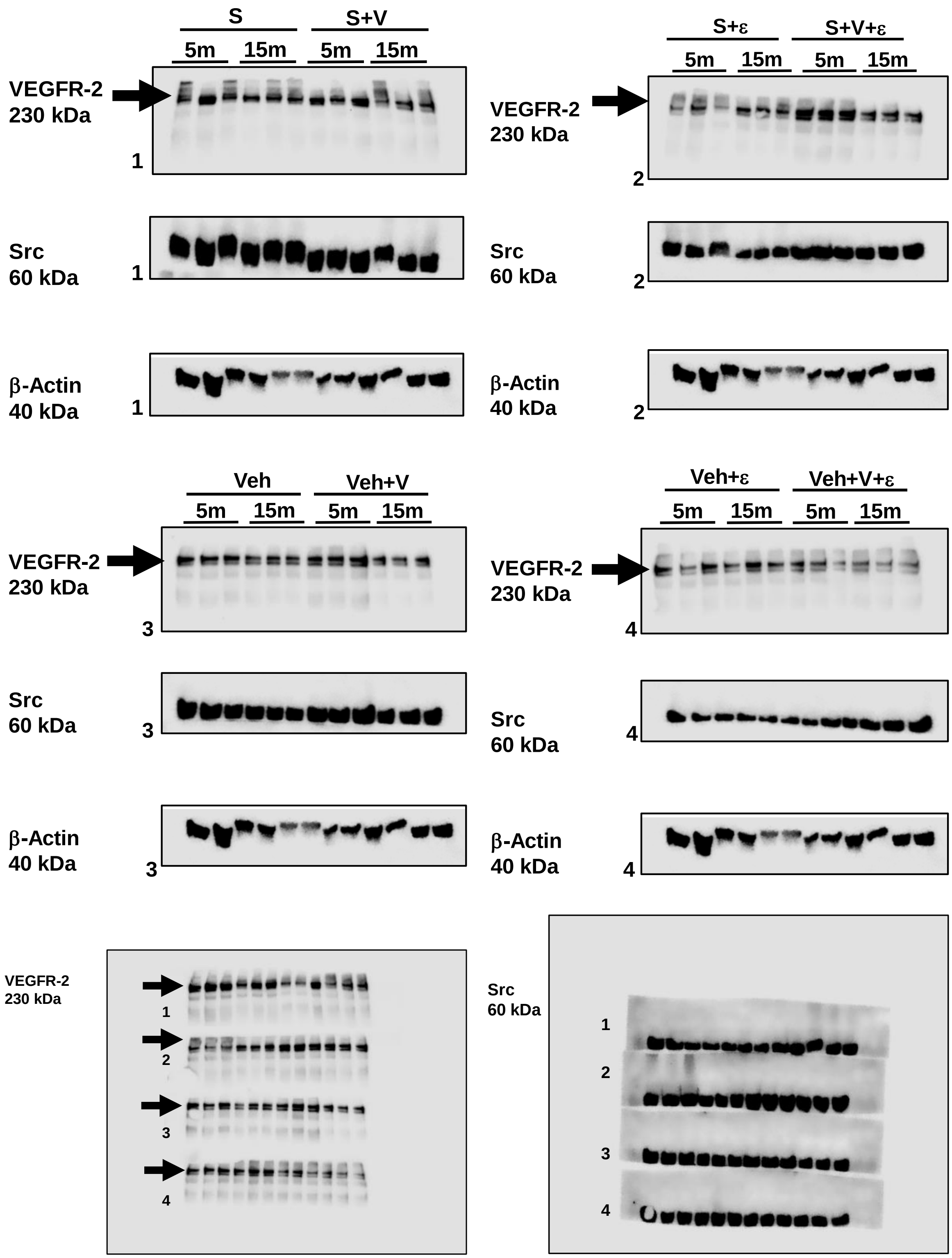

S
S+V
5m
15m
5m
15m
VEGFR-2
230 kDa
1
Src
60 kDa
1
β-Actin
40 kDa
1
S+ε
S+V+ε
5m
15m
5m
15m
VEGFR-2
230 kDa
2
Src
60 kDa
2
β-Actin
40 kDa
2
Veh
Veh+V
5m
15m
5m
15m
VEGFR-2
230 kDa
3
Src
60 kDa
3
β-Actin
40 kDa
3
Veh+ε
Veh+V+ε
5m
15m
5m
15m
VEGFR-2
230 kDa
4
Src
60 kDa
4
β-Actin
40 kDa
4
VEGFR-2
230 kDa
1
2
3
4
Src
60 kDa
1
2
3
4

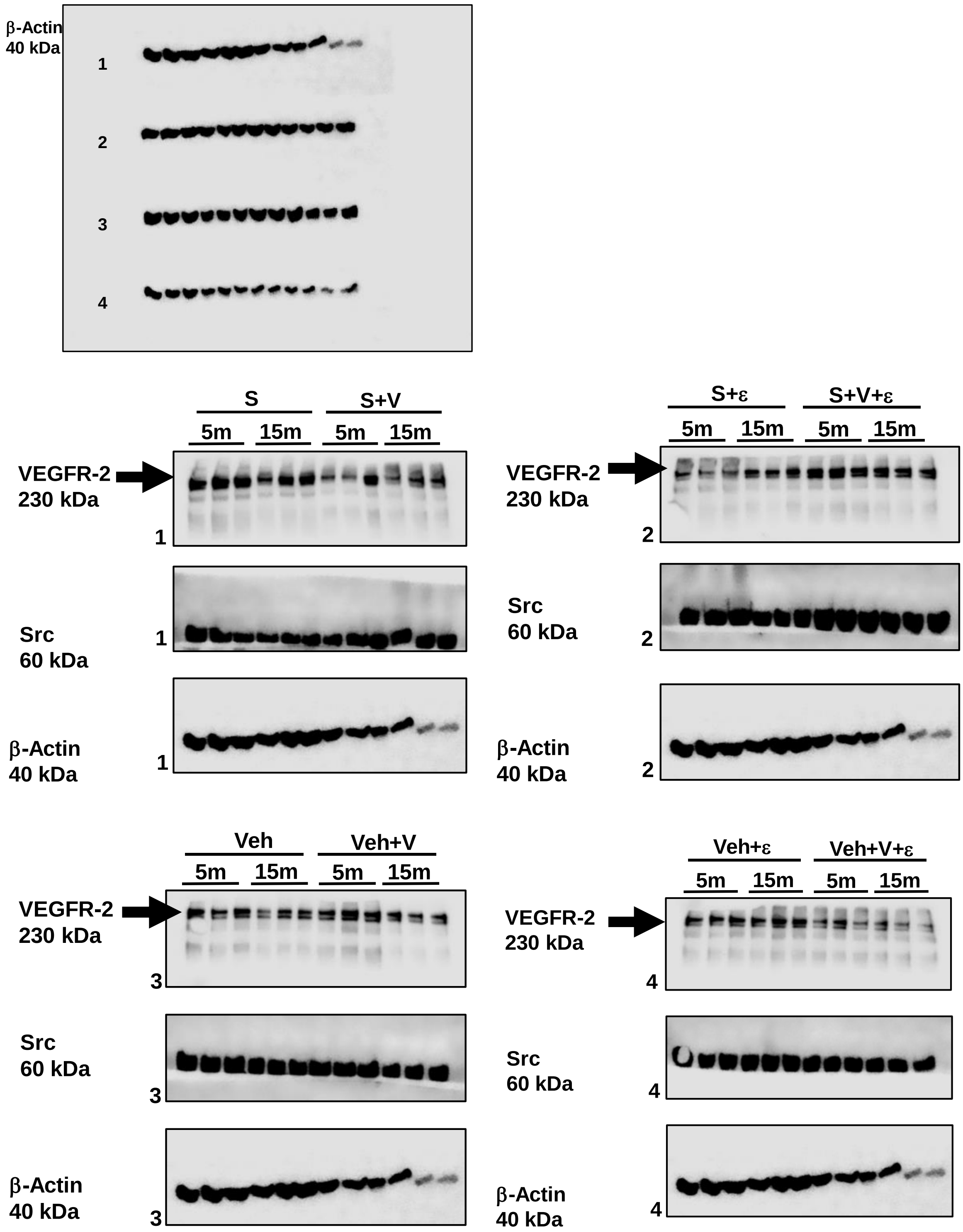

β-Actin
40 kDa
1
2
3
4
S
S+V
5m
15m
5m
15m
VEGFR-2
230 kDa
Src
60 kDa
β-Actin
40 kDa
S+ε
S+V+ε
Veh
Veh+V
Veh+ε
Veh+V+ε

4f

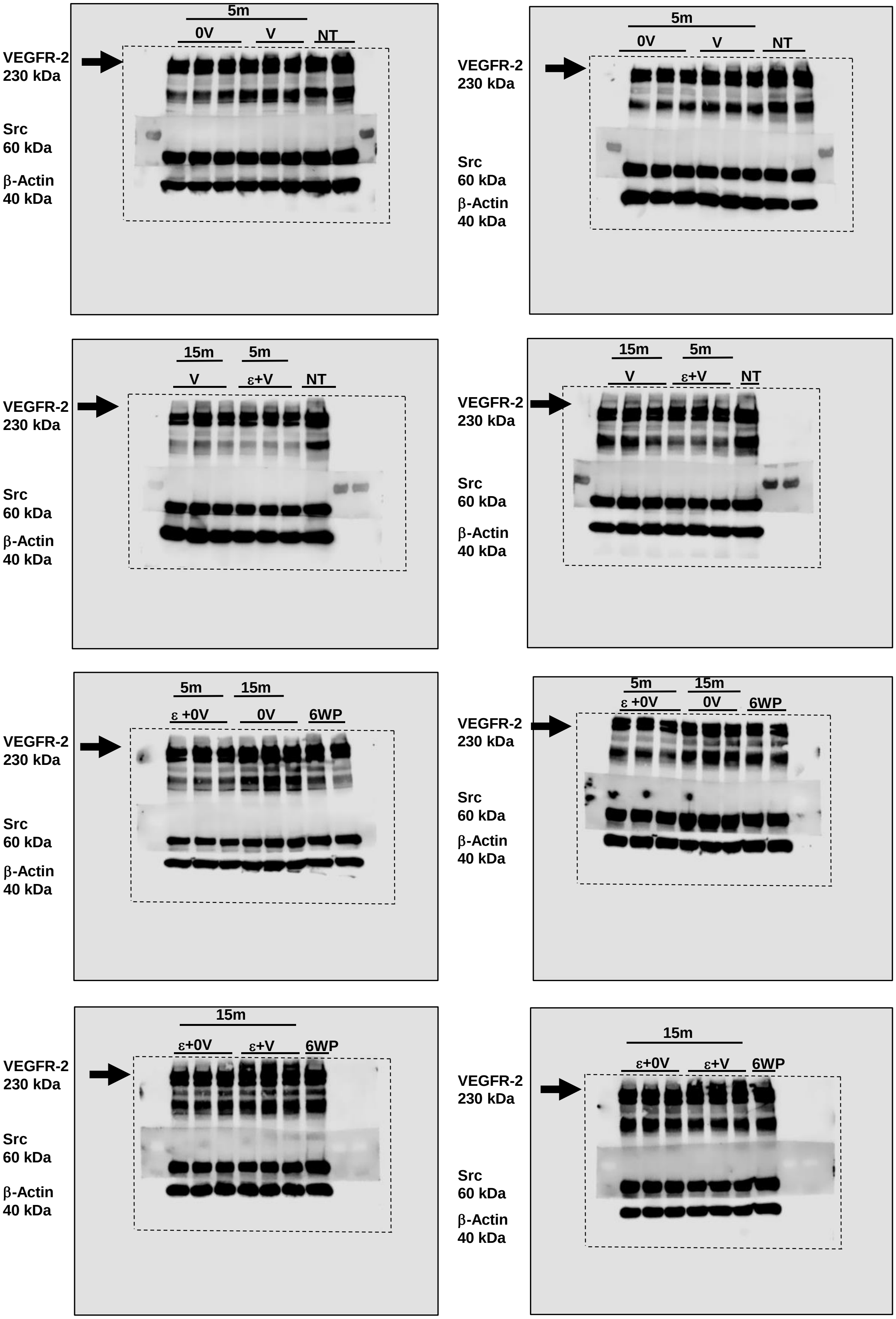
5m
0V
V
NT
VEGFR-2
230 kDa
Src
60 kDa
β-Actin
40 kDa
5m
0V
V
NT
VEGFR-2
230 kDa
Src
60 kDa
β-Actin
40 kDa
15m
5m
V
ε+V
NT
VEGFR-2
230 kDa
Src
60 kDa
β-Actin
40 kDa
15m
5m
V
ε+V
NT
VEGFR-2
230 kDa
Src
60 kDa
β-Actin
40 kDa
5m
15m
ε +0V
0V
6WP
VEGFR-2
230 kDa
Src
60 kDa
β-Actin
40 kDa
5m
15m
ε +0V
0V
6WP
VEGFR-2
230 kDa
Src
60 kDa
β-Actin
40 kDa
15m
ε+0V
ε+V
6WP
VEGFR-2
230 kDa
Src
60 kDa
β-Actin
40 kDa
15m
ε+0V
ε+V
6WP
VEGFR-2
230 kDa
Src
60 kDa
β-Actin
40 kDa

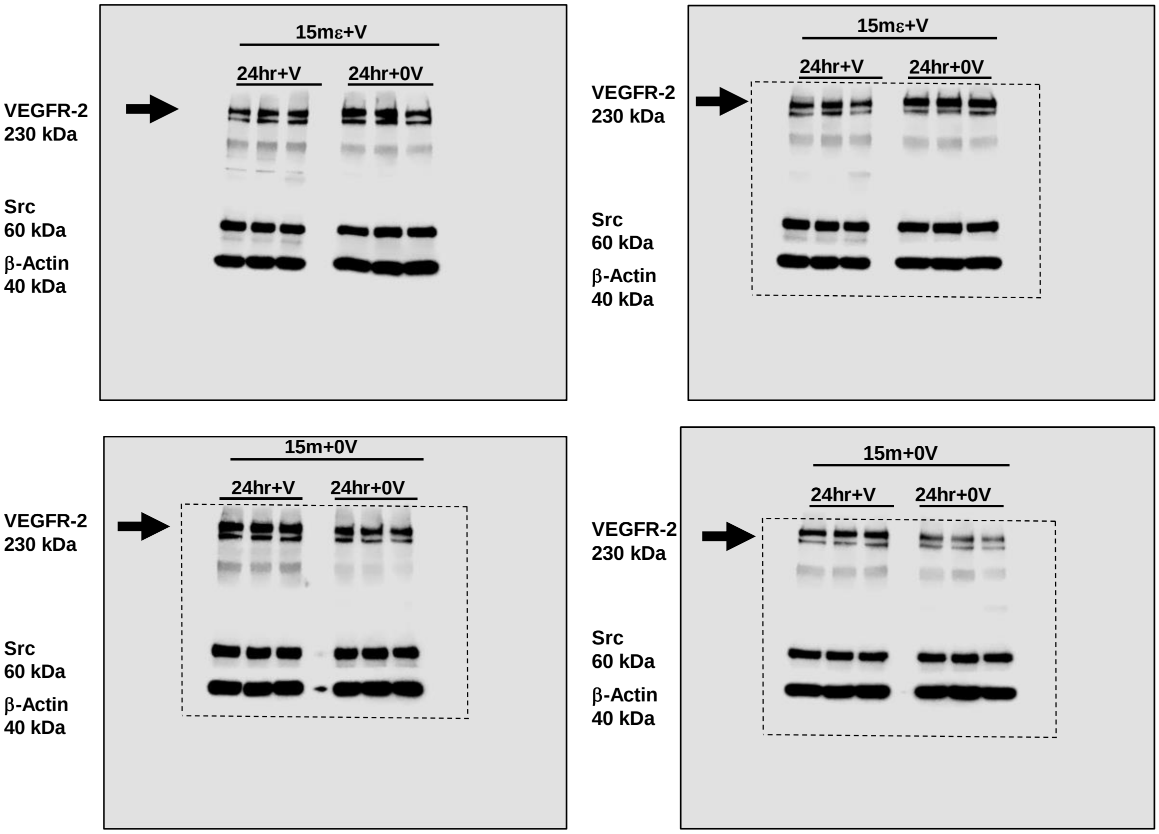
15mε+V
24hr+V
24hr+0V
VEGFR-2
230 kDa
Src
60 kDa
β-Actin
40 kDa
15mε+V
24hr+V
24hr+0V
VEGFR-2
230 kDa
Src
60 kDa
β-Actin
40 kDa
15m+0V
24hr+V
24hr+0V
VEGFR-2
230 kDa
Src
60 kDa
β-Actin
40 kDa
15m+0V
24hr+V
24hr+0V
VEGFR-2
230 kDa
Src
60 kDa
β-Actin
40 kDa

4g

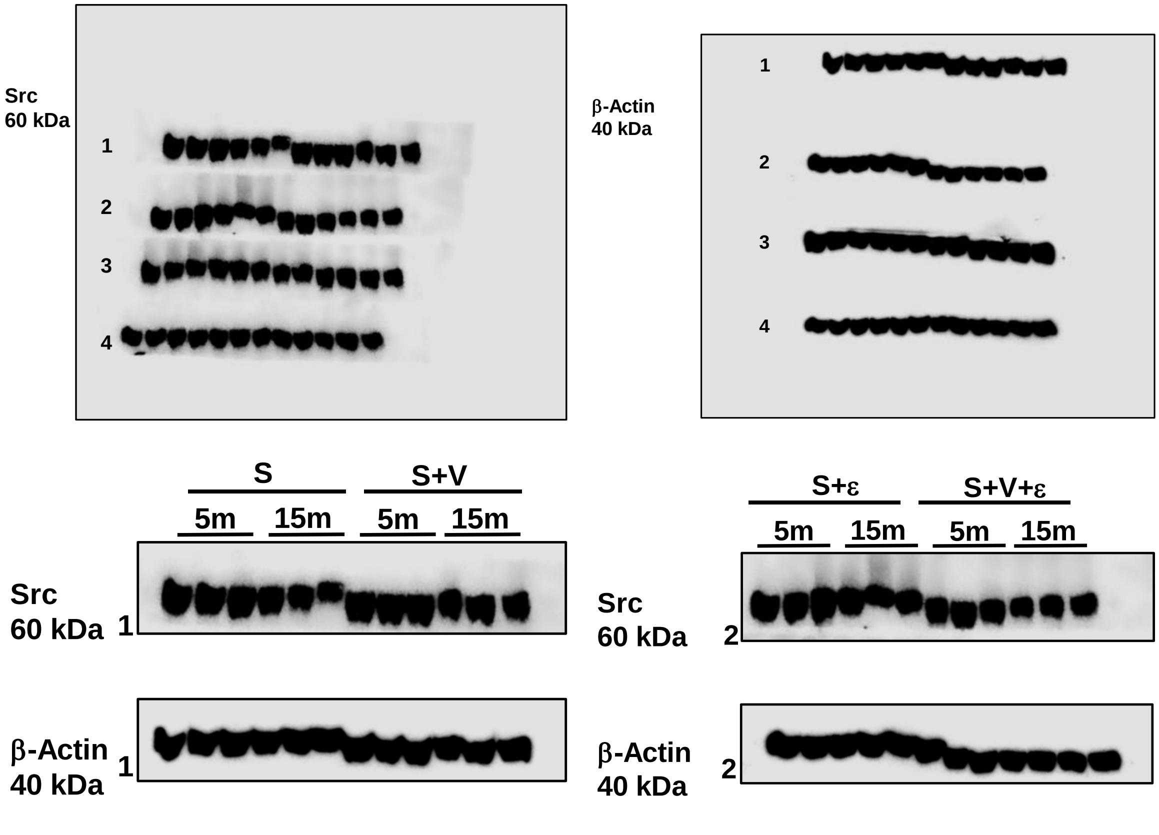
Src
60 kDa
1
2
3
4
β-Actin
40 kDa
1
2
3
4
S
S+V
5m
15m
5m
15m
Src
60 kDa
1
β-Actin
40 kDa
1
S+ε
S+V+ε
5m
15m
5m
15m
Src
60 kDa
2
β-Actin
40 kDa
2

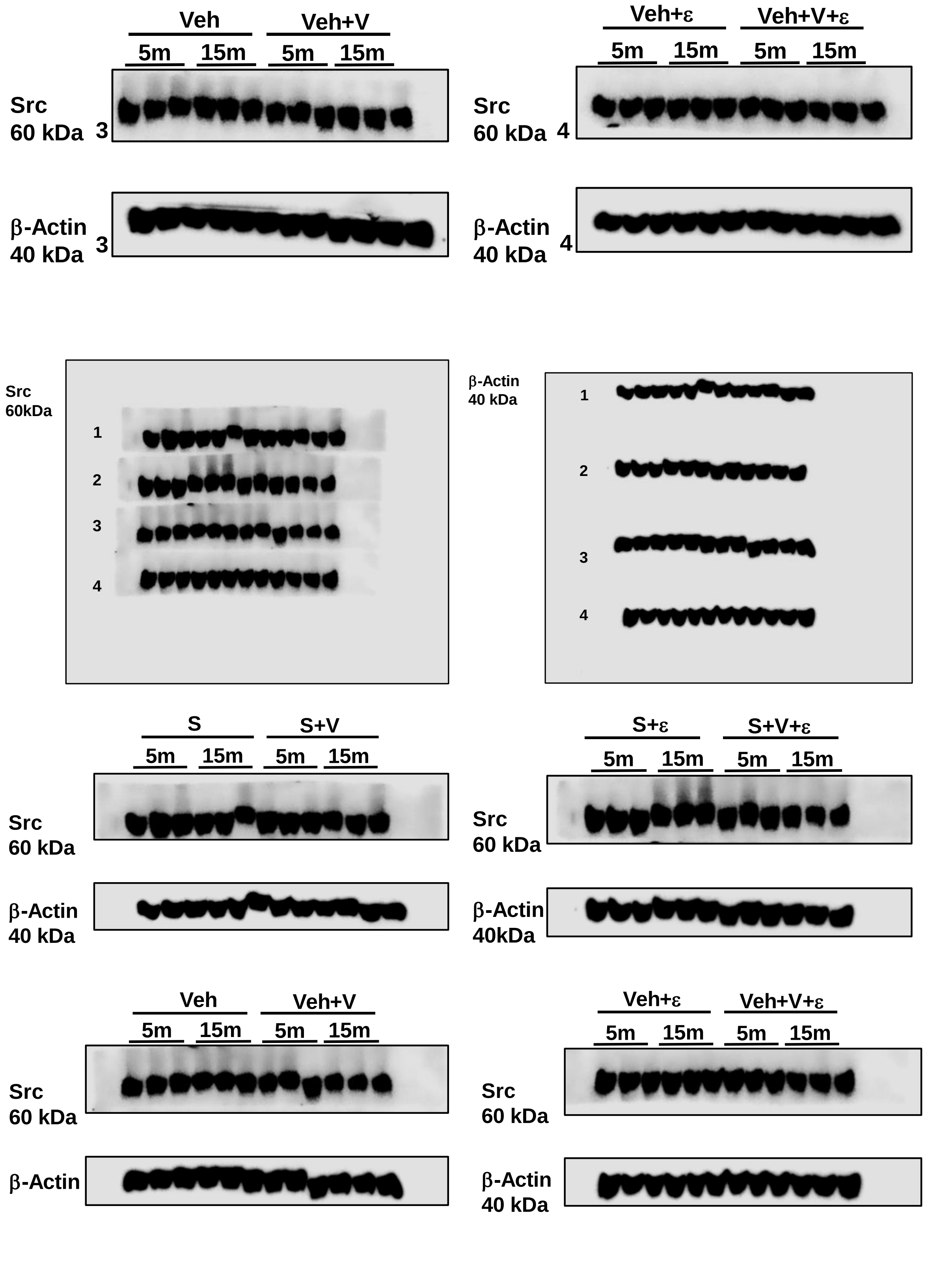

Supplemental Fig. 4 – Labelled Western Blots for Data in Fig. 3. (a) HMECs and **(b)** HUVECs were treated with control, no treatment (NT) media, VEGF (V), SU5416 (S), or both VEGF and SU5416 (V+S) for 72hr. Western blot analysis for total levels of Src was performed. **(c)** HUVECs in 3D fibrin gels with magnetic beads were treated with VEGF (V), DMSO as a vehicle control (Veh), or SU5416 (S) for 72hr. Some samples received no external magnetic stimulation while other samples were cultured above a rotating magnetic field. Src was analyzed via Western blot and normalized to

GAPDH as a loading control. **(d)** HMECs were subjected to control (NT) conditions without strain and VEGF, VEGF only (V), strain only (ε), or strain plus VEGF (ε+V) for 5min or 15min before Src analysis via Western blot. (**e**) HMECs were treated with combinations of strain, VEGF, SU5416, and a vehicle control for 5min or 15min. Samples were analyzed through Western Blot and stained for total Src. **(f-g)** Quantification of Western Blots for total Src in HUVECs with same treatment groups outlined above. Western Blots were normalized to β-actin. Arrows indicate the VEGFR-2 bands that were quantified. Full images are numbered and correspond to labelled pictures.
